# Therapeutic effectiveness of tuberculous aneurysm and risk factors for mortality: a systematic review

**DOI:** 10.1007/s11748-022-01811-9

**Published:** 2022-04-04

**Authors:** Shengwu Yi, Lingjie Sheng, Wei Li

**Affiliations:** 1 Zhejiang Hospital of Chinese Medicine, No. 54 Youdian Road, Hangzhou, Zhejiang People’s Republic of China; 2 College of Health Science, Wuhan Sports University, No. 461 Luoyu Road, Wuhan, Hubei People’s Republic of China

**Keywords:** Tuberculous aneurysm, Mycotic aneurysm, Endovascular aneurysm repair, Open aortic reconstruction, Persistent TB infection

## Abstract

**Objective:**

This study aimed to determine the therapeutic effectiveness of tuberculous aortic aneurysms (TBAAs) and the risk factors for mortality.

**Methods:**

We reviewed all case reports of TBAAs treated with open surgery or endovascular aneurysm repair (EVAR) from online database in 1996–2021. Only thoracic and abdominal aortic aneurysms were included.

**Results:**

Eighty cases of open surgery and 42 cases of EVAR were included. The 2-year mortality and perioperative mortality rates of open surgery were 11.3% and 10.0%, respectively. Emergent open surgery had a significantly higher mortality (25.0%) than non-emergent open surgery (6.7%). In the EVAR group, 2-year mortality, perioperative mortality, and TBAA-related mortality were 16.7%, 4.8%, and 10.0%, respectively. Patients with typical tuberculosis (TB) symptoms before EVAR had a significantly higher TBAA-related mortality (35.0%) than patients with no typical TB symptoms before EVAR (0%). In the open surgery group, the rate of TB recurrence (2.7% vs 2.4%) and aneurysm recurrence (8.1% vs 7.3%) were quite close between preoperative anti-TB-treated and postoperative anti-TB-treated cases. However, in the EVAR group, TB recurrence (8.7% vs 0%) and aneurysm recurrence (12.5% vs 6.25%) were more common in postoperative anti-TB-treated cases.

**Conclusion:**

Open surgery was accompanied by higher perioperative mortality, whereas EVAR was followed with higher TBAA-related mortality. Emergent surgical choices of open surgery may be associated with high perioperative mortality. Typical TB symptoms before EVAR are a significant risk factor for mortality after EVAR. Early anti-TB treatment should be administered if EVAR is the surgical option.

**Supplementary Information:**

The online version contains supplementary material available at 10.1007/s11748-022-01811-9.

## Introduction

Tuberculous aortic aneurysm (TBAA), which originates from *Mycobacterium tuberculosis* complex infection, is a rare disease with high mortality. The *M. tuberculosis* complex includes *Mycobacterium tuberculosis* (MTB), *Mycobacterium africanum*, *Mycobacterium microti*, and *M. bovis*. Bacillus Calmette–Guérin (BCG) is an attenuated strain of *M. bovis* that is widely used as a vaccine against TB infection, but it has also been used for immunotherapy for non-muscle-invasive bladder cancer [1]. Both tuberculosis (TB) infection and BCG administration can induce TBAA development. Only 41 cases of TBAA were reported in 1945–1999 with a high mortality rate of 51.2% [2]. Hershfield et al. proved that the mainstay treatment for TBAA was a combination of surgical intervention and anti-TB medications (combined therapy) [2]. It is generally accepted that surgery should be conducted immediately once TBAA is detected regardless of aneurysm size [2, 3]. For infected pseudo-aneurysms, open aortic surgery, which consists of debridement of the infected aortas and vascular reconstruction, should be the first choice. Endovascular aneurysm repair (EVAR) should be advocated in high-risk patients as a bridge or stopgap method, such as acute hemorrhage, age > 60 years, immunosuppressive status, malnutrition, weight loss, severe organ failure, and risk of aneurysm rupture [3–5]. Although open surgery is the main surgical choice, the use of EVAR has increased progressively in recent years [6]. However, the therapeutic effectiveness of open surgery and EVAR for TBAA remains unknown. The risk factors for mortality in the 2 surgical choices are also unknown. The rarity of the disease restricts the analysis of therapeutic effectiveness and risk factors in one or more centers. Therefore, here we reviewed all case reports of TBAA over the last 25 years.

## Materials and methods

**Fig. 1:**
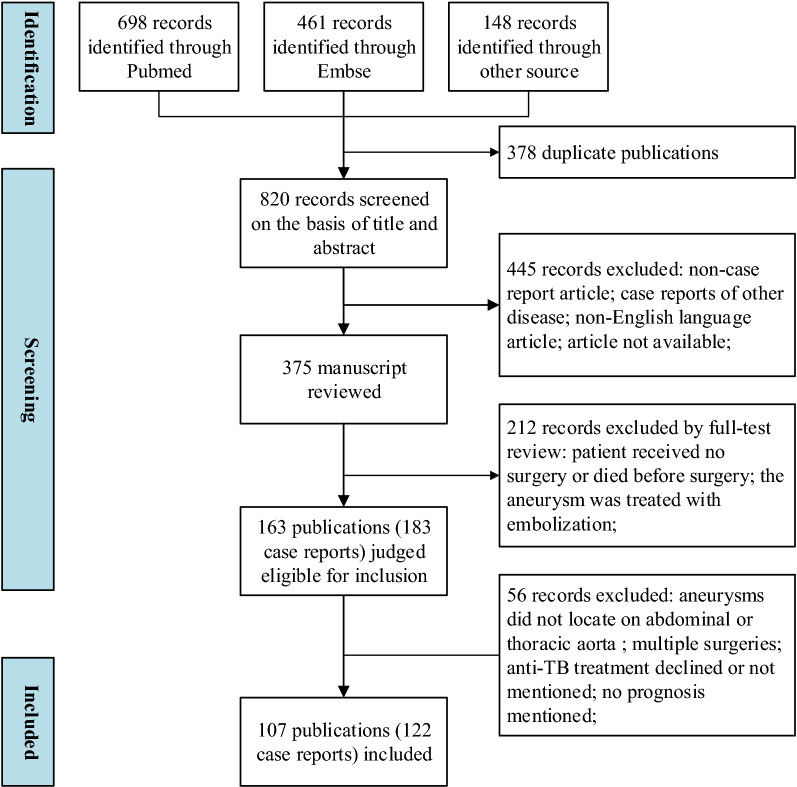
Flow diagram according to the PRISMA

This systematic review was performed according to the guidelines outlined in the Preferred Reporting Items for Systematic reviews and Meta-Analyses. The National Library of Medicine, PubMed, and Embase databases were searched using the terms “tuberculous aneurysm” or “aneurysm of tuberculosis” or “tuberculous pseudoaneurysm” or “BCG and aneurysm.” The case reports of TBAAs published in the English literature from January 1996 to September 2021 were reviewed. TBAA may develop at any site of the cardiovascular system; however, only thoracic aortic aneurysms and abdominal aortic aneurysms were included in this systematic review. The diagnosis of TBAA was made by the authors of the case reports. The inclusion criteria were patients with TBAA who underwent EVAR or open surgery. Open surgery included in this systemic review referred to arterial reconstruction with or without vascular prosthesis after aneurysm resection. EVAR refers to endovascular aneurysm repair with a stent graft rather than embolization. The exclusion criteria were as follows: (1) TBAA not located on the thoracic aorta or abdominal aorta; (2) patient having undergone multiple surgeries; (3) anti-TB treatment declined or not mentioned; and (4) prognosis not mentioned. After eligibility screening and assessment, 122 cases (80 open surgery cases and 42 EVAR cases) were included in the outcomes analysis (Fig. 1). The clinical characteristics, surgical interventions, and outcomes of each case were recorded (Online Resource 1).

Typical TB symptoms include fever with or without weight loss, anorexia, malaise, night sweats, fatigue, or a combination of other symptoms without fever. In preoperative anti-TB-treated patients, prognostic events referred to the complications that occurred after surgical interventions. In postoperative anti-TB-treated patients, the prognostic events referred to the lesions that appeared after the initiation of anti-TB treatment. In some cases, the nature of the TB was unrealized until the onset of various complications and anti-TB medications were delayed. Such complications before anti-TB treatment were not considered prognostic events.

The prognostic events analyzed in this review included death, recurrence of TB/aneurysm, surgery-associated complications, and re-interventions. Perioperative mortality was defined as death within 30 days after surgery. TBAA-related mortality was defined as death from secondary aneurysm rupture after repair within 30 days of re-intervention attributable to TBAA or death caused by TB infection. Surgery-associated complications referred to lesions directly related to the surgery, complications that occurred within 30 days of the primary procedure or re-intervention, or graft infection (non-TB origin).

### Statistical analysis

Continuous parametric data are expressed as mean and standard deviation. Categorical data are expressed as number and percentage. The chi-square test or Fisher’s exact test was used to analyze categorical data. Continuous variables were compared using Student’s t test for 2 independent samples. Kaplan–Meier estimator and Cox regression analysis were used to estimate the cumulative probability of survival. All statistical analyses were performed using the SPSS Statistics 23 (IBM). All statistical tests were 2-sided. Statistical significance was set at P < 0.05.

## Results

### Patient characteristics

A total of 122 cases (80 open surgery cases and 42 EVAR cases) were included. The diagnosis was made based on different situations: 79 cases based on culture and/or histology of the aneurysmal and peri-aneurysmal tissues; aneurysms located in the cavity of TB infection in 14 cases and aneurysms adjacent to infected organ/tissue in 4 cases; and the other 25 cases were diagnosed based on clinical deduction. The patients’ clinical characteristics are summarized in Table 1. The number of male patients was nearly 4 times that of female patients. In addition to the TB symptoms mentioned in Table 1, 20 cases presented with respiratory manifestations of TB infection, 8 cases presented with symptoms of spinal TB, 4 cases presented with symptoms of gastrointestinal TB, one case presented with symptoms of liver TB, and one case presented with symptoms of urinary TB. There were 76 case reports that have described computed tomography images of TBAA. Saccular aneurysms accounted for 82.1% and irregular aneurysms accounted for 17.9% of the aneurysms with a mean diameter of 6.3 cm (range, 2–11.9 cm). Other computed tomography findings included peri-aneurysmal mass (25.0%), stranding (6.3%), fluid (15.2%), and psoas abscess (11.8%). All 3 cases with aneurysms that ruptured during the surgical interventions were in the open surgery group. More cases in the EVAR group than in the open surgery group received preoperative anti-TB treatments.

**Table 1:** Demographic and clinical characteristics

Clinical data	Total (*n* = 122)	Open surgery (*n* = 80)	EVAR (*n* = 42)
Demographic characteristics			
Sex (male), n (%)	95 (77.9%)	67 (83.8%)	28 (66.7%)
Age (years)	54.21 ± 20.31	55.62 ± 20.11	51.52 ± 20.66
Concomitant disease			
COPD, n (%)	4 (3.3%)	4 (5.0%)	0
Hypertension, n (%)	19 (15.6%)	16 (20.0%)	37.1%)
DM, n (%)	6 (4.9%)	6 (7.5%)	0
Renal disease, n (%)	3 (2.5%)	3 (3.8%)	0
Smoke, n (%)	13 (10.7%)	8 (10.0%)	5 (11.9%)
Origin of TBAA			
TB, n (%)	89 (73.0%)	55 (68.8%)	34 (81.0%)
BCG, n (%)	33 (27.0%)	25 (31.3%)	8 (19.0%)
Location of TBAA			
Ascending aorta, n (%)	11 (9.0%)	10 (12.5%)	1 (2.4%)
Aortic arch, n (%)	11 (9.0%)	8 (10.0%)	3 (7.1%)
DTA, n (%)	33 (27.0%)	14 (17.5%)	19 (45.2%)
Suprarenal AAA, n (%)	14 (11.5%)	9 (11.3%)	5 (11.9%)
Para-renal AAA, n (%)	3 (2.5%)	2 (2.5%)	1 (2.4%)
Infrarenal AAA, n (%)	37 (230.3%)	26 (32.5%)	11 (26.2%)
AAA (not specify location), n (%)	5 (4.1%)	4 (5.0%)	1 (2.4%)
Thoraco-abdominal aorta, n (%)	8 (6.6%)	7 (8.8%)	1 (2.4%)
Rupture of TBAA			
Preoperative rupture, n (%)	55 (45.1%)	32 (40.0%)	23 (54.8%)
Rupture during surgery, n (%)	3 (2.5%)	3 (3.8%)	0
Typical TB symptoms, n (%)	71 (58.2%)	51 (63.7%)	20 (47.6%)
Fever, n (%)	68 (55.7%)	48 (60.0%)	20 (47.6%)
Weight loss, n (%)	51 (41.8%)	35 (43.8%)	16 (38.1%)
Anorexia, n (%)	19 (15.6%)	16 (20.0%)	3 (7.1%)
Fatigue, n (%)	8 (6.6%)	6 (7.5%)	2 (4.8%)
Malaise, n (%)	9 (7.4%)	7 (8.8%)	2 (4.8%)
Night seats, n (%)	15 (12.3%)	11 (13.8%)	4 (9.5%)
Symptoms of aneurysm, n (%)	22 (18.0%)	17 (21.3%)	5 (11.9%)
Pain, n (%)	78 (63.9%)	49 (61.9%)	29 (69.0%)
Emergent surgery, n (%)	38 (31.1%)	20 (25.0%)	18 (42.9%)
Anti-TB treatment			
Preoperative anti-TB treatment, n (%)	60 (49.2%)	37 (46.3%)	23 (54.8%)
Postoperative anti-TB treatment, n (%)	57 (46.7%)	41 (51.2%)	16 (38.1%)
Anti-TB (not sure), n (%)	1 (0.8%)	0	1 (2.4%)
Died before anti-TB, n (%)	4 (3.3%)	2 (2.5%)	2 (4.8%)
Duration of anti-TB, n (months)	56 (10.03 ± 4.13)	37 (10.51 ± 4.39)	19 (9.11 ± 3.48)
Duration of follow-up, n (months)	115 (16.94 ± 21.86)	75 (16.51 ± 23.63)	40 (17.74 ± 18.33)

COPD: chronic obstructive pulmonary disease; DM: diabetes mellitus; DTA: descending thoracic aorta; AAA: abdominal aortic aneurysm:

### Surgical interventions

In the 80 cases of open surgery, one patient died during the surgical procedure [7]. For the rest cases, the arteries were reconstructed with vascular grafts in 53 (67.1%) cases, autogenous veins in 2 (2.5%) cases, homografts in 2 (2.5%) cases, patch repair in 11 (13.9%) cases, and direct sutures in 6 (7.6%) cases. The repair procedure was not specified in 2 (2.5%) cases. Other 3 (3.8%) cases were repaired with 2 types of grafts or procedures for multiple aneurysms. Sixty-six (83.5%) patients underwent graft (vascular prosthesis graft, patch graft, or homograft) insertion in the open surgery group. In situ reconstruction accounted for 91.1% (72/79), while anatomical bypass accounted for 8.9% (7/79) of the repair procedures. In the 42 cases of EVAR, all TBAA were repaired using graft stents. One patient died 3 h after EVAR [8]. Detailed surgical procedures are shown in Online Resource 1.

### Outcomes

**Fig. 2:**
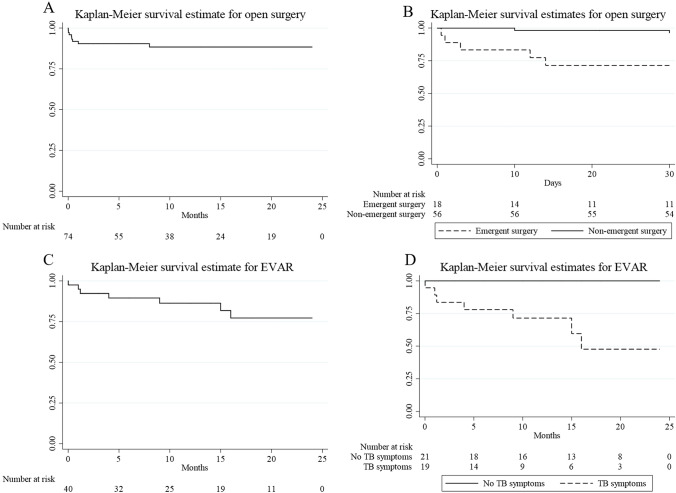
The cumulative survival curves for open surgery (**A**) and EVAR (**C**); survival analysis of emergent open surgery and non-emergent open surgery (**B**); survival analysis of patients with typical TB symptoms before EVAR and patients with no typical TB symptoms before EVAR (**D**)

All patients included in this review were treated with combined therapy. However, anti-TB medications were not administered peri-operatively in 9 cases, including 3 in the open surgery group [9–11] and 6 in the EVAR group [10, 12–16]. The delayed anti-TB treatment seemed more common in the EVAR group (15.0% [6/40]) than that in the open surgery group (3.8% [4/78]). The outcomes of all patients treated with combined therapies (All cases) and those treated with perioperative anti-TB medications (Perioperative anti-TB only) are shown in Table 2. Seven patients had a good prognosis; however, the exact follow-up duration was not specified. The reported follow-up period of the remaining cases ranged from 1 week to 10 years. Seventeen deaths were reported during follow-up. Among the cases of no reported deaths, 89.2% were followed for more than 3 months, 82.7% for more than 6 months. According to a Kaplan–Meier analysis, the 1-month survival rate was 93.0%, 1-year survival rate was 86.8%, and 2-year survival rate was 82.6%. The 2-year total mortality rate was 11.3% (9/80) in the open surgery group and 16.7% (7/42) in the EVAR group. The cumulative survival curves for open surgery and EVAR are shown in Fig. 2A and C, respectively. The detailed causes of death are shown in Table 2. Four patients died soon after surgery, even before anti-TB medications could be administered, including 2 in the open surgery group [17, 18] and 2 in the EVAR group [8, 19]. Ten patients died within 1 month after surgery: 8 in the open surgery group and 2 in the EVAR group (Table 2). Among the 8 deaths in the open surgery group, the causes of death included intraoperative aneurysm rupture [7], refractory shock [18], dissemination intravascular coagulopathy (DIC) [20], multiple organ dysfunction syndrome [17], acute respiratory distress syndrome [21], septic shock [22], aneurysm rupture [23], and unknown reason [24]. The causes of the 2 deaths within 1 month after EVAR included hypovolemia [8] and cardiac arrest [25]. In the open surgery group, 80.0% (8/10) of the deaths occurred within 1 month after open surgery. The perioperative mortality rate was higher in the open surgery group (10.0% [8/80]) than that in EVAR group (4.8% [2/42]). There were 6 deaths from 2 months to 2 years, including 1 in the open surgery group and 5 in the EVAR group. The mortality from 2 months to 2 years was significantly higher in the EVAR group than in open surgery group (12.5% vs 1.4%, p < 0.05). Four patients died of TB/aneurysm-associated complications [5, 12, 26], while 2 died of re-intervention-associated complications [19, 27]. Death caused by myocardial infarction was considered unrelated to TB/aneurysms [28]. The TBAA-related mortality rate from 2 months to 2 years was 1.4% in the open surgery group and 10.0% in the EVAR group.

**Table 2:** Outcomes after combined therapy

Outcomes in 2-year follow-up	Open surgery		EVAR	
	All cases	Perioperative anti-TB only	All cases	Perioperative anti-TB only
**Deaths**	**9/80 (11.3%)**	**9/77 (11.7%)**	**7/42 (16.7%)**	**6/36 (16.7%)**
***Deaths within 30 days of surgery***	***8/80 (10.0%)***	***8/7710.4%)***	***2/42 (4.8%)***	***2/36 (5.6%)***
Postoperative hemorrhage	4/80 (5.0%)	4/77 (5.2%)	1/42 (2,4%)	1/36 (2.7%)
Surgery-associated complications	4/80 (5.0%)	4/77 (5.2%)	1/42 (2,4%)	1/36 (2.7%)
***Deaths from the 2nd month to 2 years***	***1/72 (1.4%)***	***1/69 (1.4%)***	***5/40 (12.5%)***	***4/36 (11.1%)***
**TBAA-related death**	***1/72 (1.4%)***	***1/69 (1.4%)***	**4/40 (10.0%)**	***3/36 (8.3%)***
TB/aneurysm-associated complications	0	0	3/40 (7.5%)	2/36 (5.6%)
Complications within 30 days of re-intervention	*1/72 (1.4%)*	*1/69 (1.4%)*	1/40 (2.5%)	1/36 (2.8%)
**TBAA-unrelated death**	0	0	1/40 (2.5%)	1/36 (2.8%)
**Complications**	**19/78 (24.4%)**	**18/75 (24.0%)**	**9/40 (22.5%)**	**7/34 (20.6%)**
***Recurrence of TB/aneurysm***	***8/78 (10.3%)***	***7/75 (9.3%)***	***5/40 (12.5%)***	***3/34 (8.8%)***
**Recurrence of aneurysm**	***6/78 (7.7%)***	**5/75 (6.7%)**	***3/40 (7.5%)***	**1/34 (2.9%)**
Original TBAA increase/rupture	4/78 (5.1%)	4/75 (5.3%)	*3/40 (7.5%)*	1/34 (2.9%)
New TBAA	2/78 (2.6%)	1/75 (1.3%)	0	0
**Postoperative TB infection**	**2/78 (2.6%)**	**2/75 (2.7%)**	**2/40 (5.0%)**	**2/34 (5.8%)**
Persistent TB infection	0	0	1/40 (2.5%)	1/34 (2.2%)
Recurrence of TB infection	2/78 (2.6%)	2/75 (2.7%)	1/40 (2.5%)	1/34 (2.2%)
***Surgery-associated complications***	***11/78 (14.1%)***	***11/75 (14.7%)***	***5/40 (12.5%)***	***4/34 (11.8%)***
Complications directly related to surgery	8/78 (10.3%)	8/75 (10.6%)	3/40 (7.5%)	3/34 (8.9%)
Complications within 30 days of surgery	2/78 (2.6%)	3/75 (4.0%)	1/40 (2.5%)	1/34 (2.9%)
Graft infection (not TB)	1/78 (1.3%)	1/75 (1.3%)	1/40 (2.5%)	0
**Re-intervention**	***6/78 (7.7%)***	**6/75 (8.0%)**	***2/40 (5.0%)***	**2/34 (5.8%)**
***EVAR***	*1/78 (1.3%)*	1/75 (1.3%)	*0*	*0*
***Open surgery***	*5/78 (6.4%)*	5/75 (6.7%)	*2/40 (5.0%)*	2/34 (5.8%)

The bold values are the total number of the detailed catogories shown below them

Deaths before anti-TB treatment were not included in the complication analysis. The clinical data of the remaining 118 cases rarely changed, so we did not specify them again (Table 1). The recurrence TB/aneurysm was noted in 10.3% (8/78) of the cases in the open surgery group and 12.5% (5/40) in the EVAR group. Approximately 87.5% (7/8) of the recurrences occurred within 1 year after open surgery and 80.0% (4/5) of the recurrences occurred within 1 year after EVAR. In the open surgery group, the original aneurysm increase/rupture occurred in 4 cases [23, 27, 29, 30], while newly developed aneurysms occurred in 2 cases [10]. In both cases with newly developed aneurysms, the aneurysms were located at the proximal anastomoses of the grafts. Recurrence of TB (2.7% vs 2.4%) and recurrence of aneurysm (8.1% vs 7.3%) were quite similar between preoperative anti-TB-treated cases and postoperative anti-TB-treated cases in the open surgery group. In the EVAR group, no newly developed aneurysm was reported, but the original aneurysm increase/rupture was reported in 3 cases [5, 12, 16]. TB recurrence was reported in 4 cases after surgical intervention, including 2 in the open surgery group [31, 32] and 2 in the EVAR group [15, 16]. All postoperative TB infections occurred within 6 months after the surgical intervention. One case of persistent TB infection occurred after EVAR [15]. Recurrence of TB (8.7% vs 0) and recurrence of aneurysm (12.5% vs 6.25%) were more common in postoperative anti-TB-treated cases than in preoperative anti-TB-treated cases in the EVAR group.

Surgery-associated complications were reported in 16 patients after surgical intervention for TBAA. Complications directly related to surgery were observed in 8 cases after open surgery, acute renal dysfunction after abdominal aortic aneurysm (AAA) repair [22, 24, 32, 33], air leaks and blood clots of the bronchi after aortic arch repair [34], DIC [20], refractory shock [18], chylothorax, and gastrointestinal bleeding [35] after AAA repair. In the EVAR group, surgery directly related complications occurred in 4 cases, cerebral infarction due to occlusion of the left subclavian artery after stenting of the proximal descending thoracic aorta (DTA) [36], stent graft infection (not TB) 7 months after EVAR for the DTA [12], postoperative paraplegia due to ischemic myelopathy [37], and postoperative paralytic ileus [38]. Other complications within 30 days of surgery, such as pneumonia [39] and ARDS [21], were non-specific.

In the open surgery group, the surgical choices for re-intervention included open surgery and EVAR. One man underwent open surgery for infrarenal AAA and DTA, but the aneurysm increased in size 1 year later and was replaced with a Dacron tube graft [40]. A man with an aneurysm in the ascending aorta underwent open surgery, and the aneurysm recurred 8 months later. He underwent reoperation but died of an air embolism-induced massive cerebral infarct [27]. A man underwent open surgical repair for an infrarenal AAA, but perforation or anastomotic leak occurred after 7 days that required another open surgery [24]. A man underwent open surgical repair for an infrarenal AAA, and a false aneurysm occurred in the infrarenal region near the proximal anastomosis of the Dacron tube graft 6 months later; thus, the Dacron tube graft was removed and replaced by the deep femoral vein [10]. A man underwent open surgical repair of an infrarenal AAA; 23 months later, a false aneurysm developed near the proximal anastomosis of the Dacron tube graft for which graft explantation and replacement with a homograft were performed [10]. These re-interventions were mainly aimed at repairing recurrent TBAA. However, when recurrence of TB/aneurysm occurred after EVAR, no patient chose EVAR as the re-intervention method. A man with AAA underwent EVAR, but an AAA infection associated with iliopsoas abscess occurred 6 months later for which open surgery was performed [16]. A man with an AAA underwent EVAR, but a psoas abscess around the aorta persisted, requiring graft removal [15]. The re-interventions were mainly aimed at containing the TB infection in the EVAR group.

### Risk factors for mortality

Univariate and multivariate Cox proportional hazard regression models were used to determine prognostic factors for mortality. The possible risk factors for mortality are listed in Table 3. In open surgery-treated cases, the incidence of emergent open surgery and suprarenal AAA differed significantly between the death and non-death groups. The mortality of emergent open surgery (25.0% [5/20]) was significantly higher than that of non-emergent open surgery (6.7% [4/60]) (Fig. 2B). In addition, the mortality of suprarenal AAA (33.3% [3/9]) was higher than that of other locations (8.5% [6/71]). Multivariate analyses demonstrated that emergent open surgery was the risk factor for mortality in open surgery group after adjusting for prognostic factors. In EVAR-treated patients, there were significant differences in chronic obstructive pulmonary disease (COPD), hypertension, and typical TB symptoms between the death and non-death groups. The death group included more cases with COPD (11.1% vs 0%, *p* < 0.05), hypertension (28.6% vs 2.9%, *p* < 0.05), and typical TB symptoms (100.0% vs 37.1%, *p* < 0.05) than the non-death group. Multivariate analyses demonstrated that typical TB symptoms before EVAR were the only risk factors for mortality in EVAR-treated cases after adjusting for prognostic factors. The TBAA-related mortality rate was 35.0% (7/20) in patients with typical TB symptoms before EVAR, but no deaths were reported in patients with no typical TB symptoms before EVAR (Fig. 2D).

**Table 3:** Possible risk factors for mortality in open surgery group and EVAR group

Possible risk factors	Open surgery (*n* = 80)			EVAR (*n* = 42)		
	Non-deaths (*n* = 71)	Deaths (*n* = 9)	P value	Non-deaths (*n* = 35)	Deaths (*n* = 7)	P value
Demographic Characteristics						
Male sex, n (%)	58 (81.7%)	9 (100.0%)	0.161	22 (62.9%)	6 (85.7%)	0.277
Age (years)	55.80 ± 19.33	54.22 ± 26.86	0.826	51.29 ± 20.30	52.71 ± 24.05	0.870
Concomitant disease						
COPD,	4 (5.6%)	0	0.465	0	1 (11.1%)	0.016
Hypertension	14 (19.7%)	2 (22.2%)	0.860	1 (2.9%)	2 (28.6%)	0.016
DM	6 (8.5%)	0	0.365	0	0	–-
Renal disease	2 (2.8%)	1 (11.1%)	0.217	0	0	–-
Smoke	8 (11.3%)	0	0.288	4 (11.4%)	1 (14.3%)	0.831
TB/BCG origin			0.886			0.482
TB	49 (69.0%)	6 (66.7%)		29 (82.9%)	5 (71.4%)	
BCG	22 (31.0%)	3 (33.3%)		6 (17.1%)	2 (28.6%)	
Location of aneurysm						
Ascending aorta	8 (11.3%)	2 (22.2%)	0.349	1 (2.9%)	0	0.651
Aortic arch	7 (9.9%)	1 (11.1%)	0.906	3 (8.6%%)	0	0.421
DTA	14 (19.7%)	0	0.142	16 (45.7%)	3 (42.9%)	0.890
Suprarenal AAA	6 (8.5%)	3 (33.3%)	0.026	4 (11.4%)	1 (14.3%)	0.831
Para-renal AAA	2 (2.8%)	0	0.610	1 (2.9%)	0	0.651
Infrarenal AAA	23 (32.4%)	3 (33.3%)	0.955	8 (22.9%)	3 (42.9%)	0.272
Thoraco-abdominal aorta	7 (9.9%)	0	0.324	1 (2.9%)	0	0.651
TB symptoms	44 (62.0%)	7 (77.8%)	0.634	13 (37.1%)	7 (100.0%)	0.002
Rupture	29 (40.8%)	5 (55.6%)	0.400	19 (54.3%)	4 (57.1%)	0.890
Emergent surgery	15 (21.1%)	5 (55.6%)	0.025	16 (45.7%)	2 (28.6%)	0.403
Anti-TB treatment			0.004			0.009
Preoperative anti-TB	32 (45.1%)	5 (55.6%)		19 (54.3%)	4 (57.1%)	
Postoperative anti-TB	39 (54.9%)	2 (22.2%)		15 (42.9%)	1 (14.3%)	
Duration of anti-TB (months)	10.64 ± 4.38	–	–	8.65 ± 3.37	–	–

## Discussion

Although we know that TBAA may develop after TB infection or BCG administration, it is not easy to realize an aneurysm is of TB origin. The diagnosis of TBAA was not easy to make in clinical practice. This systematic review found that the authors made the diagnosis based on: (1) culture and/or biopsy of the aneurysmal tissue; (2) the aneurysm adjacent to the site of TB infection; and (3) clinical deduction. These diagnostic criteria may be useful guidelines in future clinical practice. There may be some differences between TB-induced aneurysms and BCG-induced aneurysms, such as patient age, duration needed for aneurysm development, and rupture rate [6]. However, most clinical features and therapeutic strategies are similar. Therefore, here we analyzed the therapeutic effectiveness of both TB- and BCG-induced aneurysms together. The 2-year mortality of TBAA after EVAR was 16.7% in this review, which coincided with the mortality of mycotic aortic aneurysms (MAA) after EVAR (17.8%) [41]. The incidence of complications after EVAR for TBAA (22.5%) was consistent with that of MAA after EVAR (24–37%) [42]. The incidences of complications and mortality were nearly the same between TBAA and MAA after EVAR. This may indicate that the therapeutic effectiveness of EVAR did not differ between TBAAs and MAAs. Although the etiology differed, TBAA may not differ from other infection-induced aneurysms in its absence.

The incidence of complications was nearly the same between the open surgery group and the EVAR group (24.4% vs 22.5%) (Table 2). However, if the patients with no perioperative anti-TB treatment were excluded, the incidence was slightly higher in the open surgery group than in the EVAR group (24.0% vs 20.6%) (Table 2). Therefore, perioperative anti-TB treatment may affect complications. The major concern of intervention for TBAA was TB/aneurysm recurrence. The incidence of TB/aneurysm recurrence was quite close between the open surgery and EVAR groups (10.3% vs 12.5%) after combined therapy (Table 2). This may indicate that surgical choice does not have an effect on the postoperative recurrence of TB/aneurysms. However, in more detail, newly developed aneurysms were only reported in the open surgery group, and both cases had newly developed aneurysms located at the proximal anastomoses of grafts [10, 43]. It is unclear why all of these newly developed aneurysms were located at the proximal anastomoses of the grafts. The insufficient debridement of the infected aorta may be associated with the coincidence to some extent. Recurrence of TB (2.7% vs 2.4%) and aneurysm (8.1% vs 7.3%) were quite similar between preoperative anti-TB-treated cases and postoperative anti-TB-treated cases in the open surgery group. This implies that anti-TB medications could be postponed for a while if the infected tissues are debrided. However, the recurrence of TB (8.7% vs 0%) and recurrence of aneurysms (12.5% vs 6.25%) were more common in postoperative anti-TB-treated cases than preoperative anti-TB-treated cases in the EVAR group. In addition, persistent TB infection was reported only in the EVAR group. Thus, anti-TB medications may be better administered preoperatively if EVAR is the surgical choice. Some authors have suggested that anti-TB treatments should begin at or shortly after surgery, and good outcomes may be anticipated [2]. Others insisted that early anti-TB treatment should be recommended to reduce postoperative complications and mortality [44]. In open surgery-treated cases, anti-TB treatment can be administered shortly after surgery. However, early anti-TB treatment should be administered if EVAR is selected. Moreover, the TB nature was more prone to be neglected in EVAR, as delayed TB detection was more common in EVAR group than in open surgery group (15.0% vs 3.8%). This may have resulted in more cases of recurrent TB/aneurysm in patients treated with EVAR.

Researchers have shown that, for common AAA, the re-intervention rate was significantly higher in the EVAR group [45]. For MAA, infection-related re-intervention was more common in the EVAR than the open surgery group at mid-term [42]. In this systematic review, the re-intervention rate was quite similar between open surgery and EVAR (7.7% vs 5.0%). However, the re-intervention was mainly aimed at repairing the recurrent aneurysms in the open surgery group and containing postoperative TB infection in the EVAR group (Table 4).

**Table 4:** Recurrence of TB/aneurysm and re-intervention

Recurrence of TB/aneurysm	Open surgery (*n* = 78)		EVAR (*n* = 40)	
	Recurrence	Re-intervention	Recurrence	Re-intervention
Original aneurysm increase/rupture	4	3	3	1
New aneurysm	2	2	0	0
Persistent TB infection	0	0	1	1
Recurrence of TB infection	2	1	1	1

EVAR simplifies the surgery procedure and may theoretically provide better outcomes. But the fact was that, for common (non-mycotic) AAAs, EVAR had greater aneurysm-related and total mortality than open surgery in late follow-up [45]. For MAA, EVAR benefited short-term survival (6 months) but not long-term survival compared with open surgery [46, 47]. The results of this systematic review are consistent with these conclusions. In this review, although more patients in the EVAR group received preoperative anti-TB treatments, the TBAA-related mortality rate was still higher in the EVAR group. Compared with open surgery, EVAR had higher total mortality (16.7% vs 11.3%) and TBAA-related mortality (10.0% vs 1.4%), but a lower perioperative mortality (4.8% vs 10.0%). In fact, the satisfactory outcome of most EVAR cases suggests that EVAR could serve as an alternative to open surgery; however, the risk factors must be identified. Previous studies have identified ruptured aneurysms, fever, and suprarenal AAA as risk factors for mortality after MAA treatment [42, 48]. However, this systemic review found that typical TB symptoms before EVAR were the only significant risk factors for mortality in EVAR-treated cases (Fig. 2D). In previous studies, typical TB symptoms were reported in approximately 63% of patients with TBAAs [2, 6]. In this review, 63.7% of patients treated with open surgery and 47.6% of those treated with EVAR presented with typical TB symptoms (Table 1). Open surgery should be the first choice if typical TB symptoms are observed in patients with TBAA. In this review, approximately 42.9% of the patients were given EVAR as an emergent choice, but EVAR was primarily chosen when patients were stable and their conditions were not emergent. On the contrary, 25.0% of patients were given open surgeries as emergent choice. However, emergent open surgery contributed to a high perioperative mortality (Fig. 2D). As mentioned previously, EVAR should be used instead of open surgery in high-risk patients and emergent conditions. However, it is not easy to make a surgical choice even when risk factors are identified, as the clinical conditions are often complicated. Obviously, EVAR may not be ideal in patients who are considered suitable candidates for open surgery, such as those with aorto-duodenal or arterio-cutaneous fistulas. EVAR can be a temporary measure in emergent conditions. However, open surgery should be performed if typical TB symptoms present before EVAR, TB infections persist, or other events occur after EVAR. However, if the emergent state is controlled by EVAR and there are no TB symptoms before EVAR and no TB/aneurysm-associated complications, open aortic surgery may be unnecessary. In addition, the aneurysm may rupture with shock and the typical TB symptoms manifested. Besides, the TB infection may be accompanied with weight loss and weakness of the bodies. In such conditions, the open surgery was poorly tolerated and the EVAR was at the risk of mortality. In this way, EVAR may be used as a bridge method. However, in clinical practice, the most probable challenge may be the failure to recognize the TB nature of aneurysms.

## Study limitations

Reviewing case reports retrieved from online databases may involve selection bias. In addition, some clinical data could not be obtained, as the reports did not mention all required details. The debut of TB/aneurysm recurrence is difficult to define. With limited cases and prognostic events, the precision of estimates and power may not be that high. For these reasons, we did not directly compare mortality and complications between the open surgery and EVAR groups.

## Conclusion

Open surgery had a higher perioperative mortality rate, while EVAR had higher TBAA-related and total mortality rates. However, the surgical choice of open surgery or EVAR was not appropriately made in some of the reported cases. Emergent open surgery may be associated with higher perioperative mortality. In contrast, the typical TB symptoms before EVAR are significant risk factors for mortality after EVAR. The recurrence of TB/aneurysm was unavoidable after open surgery or EVAR. However, newly developed aneurysms were more common after open surgery, especially at the site of proximal anastomoses of the grafts. In open surgery-treated cases, anti-TB treatment can be administered shortly after surgery. Whereas, early anti-TB treatment should be administered if EVAR is selected.

## Supplementary Information

Below is the link to the electronic supplementary material.

Supplementary file 1 (DOCX 198 KB)
