# Supplementary material for: Therapeutic effectiveness of tuberculous aneurysm and risk factors for mortality: a systematic review
Source: Gen Thorac Cardiovasc Surg. 2022 Apr 4;70(6):515–25. doi: 10.1007/s11748-022-01811-9 (PMC9135858; doi:10.1007/s11748-022-01811-9)
Supplement: Supplementary file 1 — (DOCX 198 KB) [file 11748_2022_1811_MOESM1_ESM.docx]

Case reports of tuberculous aneurysms that underwent open surgeries or EVARs

| Literature | Year | Sex; age | Origin of aneurysm | Site of TB | Concomitant disease | Aneurysms | Rupture | Preoperative condition | Emergent surgery | Surgery | Preoperative/postoperative anti-TB treatment | Duration of anti-TB (month) | Follow up (month) | Prognosis |
| --- | --- | --- | --- | --- | --- | --- | --- | --- | --- | --- | --- | --- | --- | --- |
| [1] | 1996 | M 68 | TB | Aneurysm tissue | No | Thoracic aorta | Rupture | Shock | Yes | Open surgery: aneurysm resection and repaired with Dacron patch | Postoperative anti-TB | Not mentioned | 12 | Recover well |
| [2] | 1996 | F 47 | TB | Not sure of infection site and aneurysm | No | DTA | No rupture | Stable | No | Open surgery: aneurysm resection and repaired with Dacron patch | Preoperative anti-TB | Not mentioned | 13 | Recover well |
| [3] | 1996 | M 80 | TB | Pott's diseas (T12 and L1), left psoas abscess | CAD | Suprarenal AAA | No rupture | Stable | No | Open surgery: the aneurysm resected and revascularized with deep vein graft | Postoperative anti-TB | 10 | 6 | Recover well |
| [4] | 1996 | M 76 | BCG | Pott's diseas (T12 and L1), right psoas abscess | CAD, myocardial infarctions | Infrarenal AAA | No rupture | Stable | No | EVAR with stent graft (Barone Buenos Aires Argentina) | Preoperative anti-TB | Not mentioned | 15 | Died of myocardial infarctions |
| [5] | 1998 | M 34 | TB | Pulmonary TB | HIV | Ascending aorta | Rupture | Stable | No | Open surgery: the aorta was replaced with hemograft | Preoperative anti-TB | Not mentioned | 3 | Recover well |
| [6] | 1998 | M 71 | BCG | Aneurysm only | Myocardial infarction and paroxysmal atrial fibrillation, bladder carcinoma | Infrarenal AAA | Rupture | Stable | No | Open surgery: aneurysm, was excised and a bifurcated polyester graft was inserted | Postoperative anti-TB | Not mentioned | 8 | No complications |
| [7] | 1999 | F 27 | TB | Pulmonary TB | No | DTA | No rupture | Nothing specific | No | Open surgery: excise the pseudoaneurysm and the defect was closed with 0Prolenesutures | Postoperative anti-TB | 10 | 10 | Paralysis of the left vocal cord |
| [8] | 1999 | M 14 | TB | Pulmonary TB | No | Suprarenal AAA | No rupture | Stable | No | EVAR with aortic graft | Die before anti-TB; |  | Died | Died from hypovolemia 3 h after EVAR |
| [9] | 1999 | M 77 | TB | Aneurysm tissue | CAD, hypertension, | AAA | Rupture | Shock | Yes | Open surgery: aorta was debrided and oversewn proximally and distally with polypropylene sutures | Die before anti-TB |  | 4 days | Expired on the fourth postoperative day from MODS |
| [10] | 1999 | M 58 | BCG | Aneurysm tisssue | Bladder cancer | AAA | Rupture | Stable | No | Open surgery: endarterectomy and Dacron bifurcated graft was placed in the bed of the resected aneurysm | Postoperative anti-TB | 12 | 132 | Recover well |
| [10] | 1999 | M 71 | BCG | Aneurysm tissue | Bladder cancer | Infrarenal AAA | No rupture | Stable | No | Open surgery: excision of the aneurysm with the placement of an aortic right common iliac artery bypass graft | Postoperative anti-TB | 12 | 48 | Recver well |
| [11] | 1999 | M 67 | TB | Pulmonary TB | No | Infrarenal AAA | Rupture | Stable | No | Open surgery: The area was debrided, and an omental patch was inserted over the aorta and iliac vessels for added protection. And an axillobifemoral graft performed. | Preoperative anti-TB; | A full course | 3 | Recover well |
| [11] | 1999 | F 77 | TB | Spinal TB (T11-12), pulmonary TB | No | Suprarenal AAA | No rupture | Stable | No | Open surgery: aneurysm resection and revascularization with a dacron aortic interposition graft | Preoperative anti-TB; | Not mentioned | 2 | Recover well |
| [12] | 2000 | F 42 | TB | Psoas abscess | Not mentioned | Suprarenal AAA | No rupture | Nothing specific | No | EVAR with three Z-segments covered with expanded polytetrafluoroethylene (Taewoong Medical, Seoul, Korea) and with the upper one-third partially fenestrated to ensure flow along the celiac axis | Preoperative anti-TB | 7 | 24 | No complications |
| [12] | 2000 | M 41 | TB | Psoas abscess | Not mentioned | Infrarenal AAA (level of inferior mesenteric artery) | Rupture | Nothing specific | Not specify | EVAR with stent graft (Vanguard Straight; Boston Scientific) through the right femoral artery | Postoperative anti-TB | Not mentioned | 18 | No complications |
| [13] | 2000 | M 38 | TB | TB cervical lymphadentitis | Not mentioned | Suprarenal AAA | Rupture | Lower gastrointestinal bleeding need blood transfusions | No | Open surgery: the aneurysm was resected, and the aortic wall defect was closed. | Preoperative anti-TB | Not mentioned | 36 | No complications |
| [14] | 2000 | M 14 | TB | Tuberculous lymphadenitis | No | Suprarenal AAA | Rupture | Stable | Yes | Open surgery: Dacron graft implantation with visceral arterial reimplantation | Preoperative anti-TB | Dead | Dead | Died on the first postoperative day for DIC |
| [15] | 2001 | M 25 | TB | TB lymphadenitis | Hypertension and cardiomyopathy | Thoracic and abdominal aorta | No rupture | Nothing specific | No | Open surgery: thoracoabdominal aneurysm resection with placement of a hemashield graft; aorta-to- left-renal-artery bypass with greater saphenous vein. | Preoperative anti-TB | Not mentioned | 12 | Renal dysfunction requires hemodialysis |
| [16] | 2001 | M 22 | TB | Pulmonary TB; cervical lymph nodes TB | No | Aortic arch | No rupture | Nothing specific | No | Open surgery: the aneurysm incised and the rent in the aortic wall repaired with a Gore-Tex patch | Preoperative anti-TB | 6 | 36 | No complications |
| [16] | 2001 | M 40 | TB | Pulmonary TB | Hypertension | DTA | No rupture | Nothing specific | No | Open surgery: debridement of aneurysm and a Vascutek graft was interposed between the proximal and distal aorta. | Postoperative anti-TB | Not mentioned | 20 | No complications |
| [16] | 2001 | M 30 | TB | Tubercular pericardial effusion | No | Ascending aorta | No rupture | Nothing specific | No | Open surgery: aneurysm was incised and opened longitudinally. The rent was directly closed with pledgetted sutures. | Preoperative anti-TB | Not mentioned | 8 | Aneurysm recurred at 8^th^ month, he was reoperated but died of air embolism induced massive cerebral infarct |
| [16] | 2001 | M 39 | TB | Pulmonary TB | No | Infrarenal AAA | No rupture | Nothing specific | Yes | Open surgery: aneurysm resected and aortic continuity established with interposition tube graft. | Preoperative anti-TB | Not mentioned | 24 | No complications |
| [16] | 2001 | M 32 | TB | Pulmonary TB | No | DTA | No rupture | Nothing specific | Yes | Open surgery: the aneurysm was incised and opened, the rent in the aortic wall was repaired with a Gore-Tex patch. | Postoperative anti-TB | 6 | 18 | No complications |
| [17] | 2001 | M 65 | BCG | Multiple organ | Granulomatous hepatitis | Infrarenal AAA and suprarenal AAA | No | Stable | No | Open surgery: Dacron-graft was placed in situ | Before anti-TB could be given | Dead | Dead | Died from shock a few hours ago as the shock was refractory |
| [18] | 2002 | F 55 | TB | Liver TB | DM, hypertension, CKD hemodialysis, | DTA | Rupture | Nothing specific | Not specify | Open surgery: the affected area debrided and the aorta repaired with a Dacron graft and wrapped with a piece of omentum. | Preoperative anti-TB | Not mentioned | 6 | No complications |
| [19] | 2002 | M 59 | TB | Intracranial tuberculoma | Not mentioned | Ascending aorta | No rupture | Nothing specific | No | Open surgery: the distal portion of the ascending aorta clamped and incised, Dacron prothesis was sewn into the distal end | Preoperative anti-TB | Not mentioned | 36 | No complications |
| [20] | 2003 | F 65 | TB | Aneurysmal contents and aortic wall | Hypertension; DM | Distal aortic arch | Rupture | Nothing specific | No | Open surgery: the aneurysm dissected and repaired using a patch of aortic homograft. | Postoperative anti-TB | 12 | 6 | Air leak and blood clots of left upper lobe bronchi after surgery |
| [21] | 2003 | M 52 | TB | Pulmonary TB | No | Aortic arch | Rupture during surgery | Nothing specific | No | Open surgery: During dissection of the proximal aortic arch, the aneurysm ruptured, with massive cataclysmic hemorrhage | Preoperative anti-TB | Dead | Die during the surgery | Died of aneurysm rupture induced cataclysmic hemorrhage |
| [22] | 2003 | M 53 | TB | Aortic wall | Not mentioned | Ascending aorta | Rupture | Cardiac arrest and CPR; ventilation | Yes | Open surgery: aneurysm resected and replaced with a synthetic graft | Postoperative anti-TB | 9 | 19 | No complications |
| [23] | 2003 | M 54 | TB | Miliary TB | Chronic silicotuberculosis; smoke and drinking | Aortic arch | No rupture | Nothing specific | No | Open surgery: aneurysm repaired with a Dacron patch and a running suture; | Preoperative anti-TB | Not mentioned | 36 | No complications |
| [23] | 2003 | M 72 | TB | Aneurysm only | Deteriorating cardiopulmonary status | Aortic arch | Rupture | Deteriorating cardiopulmonary status ventilation; | Yes | Open surgery: total replacement of the aortic arch with elephant trunk extension of the tubular graft; | Postoperative anti-TB | 12 | 24 | No complications |
| [24] | 2003 | F 68 | TB | Multiple organ | No | DTA | Rupture | Severe hematemesis and hemoptysis and anemia | Yes | Open surgery: descending thoracic aorta was replaced with Hemashield Gold (Meadox Medicals Inc, Oakland, NJ) under the shunt using a THI Aortic Perfusion Cannula (Argyle, St. Louis, MO) | Postoperative anti-TB | 6 | 13 | No complications |
| [25] | 2004 | M 33 | TB | Pulmonary TB | No | Suprarenal AAA | No rupture | Nothing specific | No | Open surgery: periaortic tissue and aortic wall were debrided, and repair of the aneurysm with a synthetic patch. | Preoperative anti-TB | 18 | 9 | No complications |
| [26] | 2005 | M 61 | TB | Miliary TB spinal TB | Spondylodiscitis | Thoracoabdominal aorta | No rupture | Nothing specific | No | Open surgery: affected vessel segment resected, reconstruction with aortic homograft | Preoperative anti-TB | 12 | 15 | Discitis (T2 and T3) may be induced by TB occurred at 6th months and anti-TB restarted; peripheral neurologic deficit |
| [27] | 2005 | M 76 | TB | Miliary TB; pulmonary TB; mesenteric TB lymphadenitis | Epididymitis | Infrarenal AAA | Rupture | Nothing specific | No | Open surgery: aneurysm resected and continuity was restored with prosthesis | Postoperative anti-TB | Not mentioned | 6 | No complications |
| [28] | 2005 | M 69 | BCG | Spinal TB with abscess formation | Bladder carcinoma; surgery for appendicitix and colon carcinoma | Infrarenal AAA | Rupture | Nothing specific | Not specify | Open surgery: replaced with bifurcated vascular graft | Postoperative anti-TB | 9 | 36 | Lumber disease and left psoas abscess (not TB) more than 2 years later |
| [29] | 2005 | M 63 | TB | Multiple organ | Hypertensive | Aortic arch and DTA | No | Stable | No | Open surgery: surgical resection of the aneurysm on arch and interposition of a tube graft; thoracic aneurysm was replaced from the arch down to the descending aorta | Postoperative anti-TB | 18 | 28 | Died from unknown reasons |
| [30] | 2006 | M 79 | TB | Spinal TB | Chronic low back pain | Infrarenal AAA | No rupture | Nothing specific | No | Open surgery: aneurysmectomy endarterectomy aorta, placement of a synthetic bypass graft | 3 weeks postoperative anti-TB | Not mentioned | 3 | Decreased motor strength of the lower limbs |
| [31] | 2007 | M 68 | TB | Pulmonary TB | Smoke, angina, pulmonary insufficiency, hypertension | DTA | Rupture | Nothing specific | Yes | EVAR with stent graft (Talent, Medtronic Inc, Minneapolis, Minn) under general anesthesia | Preoperative anti-TB | 12 | 16 | Die from aneurysm rupture |
| [32] | 2007 | M 63 | TB | Pulmonary TB | Smoke, vascular disease bilateral popliteal to dorsalis pedis bypass | Proximal DTA | Rupture | Intubation | Not specify | EVAR with a graft (Zenith TX2 TAA,Cook Inc, Bloomington, IN, USA) through upper midline laparotomy | Postoperative anti-TB | Not mentioned | 6 | Bilateral cerebellar and left occipital infarcts due to occlusion of left subclavian artery |
| [33] | 2007 | M 80 | BCG | BCG-osis Aneurysm wall | Bladder carcinoma; hypertension, COPD, smoke | Infrarenal AAA | Rupture | Aneurysm ruptured | Not sure | Open surgery: debridement of infected tissues reconstructed aorta with Dacron graft soaked in rifampin | Postoperative anti-TB | 12 | 15 | Postoperative pneumonia |
| [34] | 2007; | M 27 | TB | Spinal TB, psoas abscess | Destruction of the vertebra | DTA and proximal AAA | No rupture | Nothing specific | Not sure | Open surgery endoaneurysmorrhaphy of thoracoabdominal aneurysm with multiple layer suture | Preoperative anti-TB | 9 | 33 | No complication |
| [35] | 2007 | M 80 | TB | Pulmonary TB | Not mentioned | Infrarenal AAA | Rupture | Severe anemia | No specify | Open surgery: aneurysm resected, replaced with Dacron graft. | Postoperative anti-TB | Till death | 0.4 | Postoperative AKI required hemodialysis and died from septic shock 11 days after operation |
| [36] | 2007 | M 70 | TB | Spinal TB | No | DTA | No | Stable | No | Open surgery: debridement of aneurismal wall and necrotic tissues, the aneurysm was replaced with a prosthetic graft | Preoperative anti-TB | Not mentioned | 24 months | No complications |
| [37] | 2007 | M 69 | TB | Miliary tuberculosis | Interstitial pneumonia | AAA (not sure) | No | Stable | No | Open surgery: surgical resection | Postoperative anti-TB | Not mentioned | Not mentioned | Improvement of clinical findings |
| [38] | 2008 | F 34 | TB | Aneurysm and surrounded tissues | Not mentioned | Suprarenal AAA | No rupture | Nothing specific | No | Open surgery: aneurysm resection and debridement of infected tissues with polyester tube graft inserted, surrounded arteries reimplanted with aortic graft | Postoperative anti-TB | 12 | 120 | No complications |
| [38] | 2008 | M 77 | TB | The aneurysm | Not mentioned | Infrarenal AAA | No rupture | Nothing specific | No | Open surgery: debridement of infected tissues and segment of aorta, aortic replacement with double coaxial venous graft | Postoperative anti-TB | 9 | 96 | No complications |
| [38] | 2008 | F 37 | TB | The aneurysm wall | Pulmonary sarcoidosis | Infrarenal AAA | No rupture | Nothing specific | No | Open surgery: aneurysm resection, reconstruction with a polyester graft coated with collagen and silver (intervascular, La Ciotat, France). | Postoperative anti-TB | 9 | 6 | No complications |
| [39] | 2008 | M 62 | TB | Spine TB | Not mentioned | Infrarenal AAA | No rupture | Nothing specific | No | Open surgery: repair the aneurysm with an in itu silver-coated bifurcated Dacron graft | Preoperative anti-TB | 6 | 12 | No complications |
| [40] | 2008 | M 69 | TB | Pulmonary TB | Colon cancer; radical resection | AAA | Rupture | Hypovolemic shock and collapse | Yes | Open surgery: aneurysm resection and repair of the aorta with Dacron graft | Postoperative anti-TB | Not mentioned | 24 | No complications |
| [41] | 2008 | M 79 | BCG | Aneurysm | Coronary artery disease, gastroesophageal reflux disease, hypothyroidism, bladder cancer | Infrarenal AAA | No | Stable | No | Open surgery: The aneurysm  Was excised after extensive irrigation and debridement, and a right axillary to right femoral and right femoral to left femoral crossover bypass graft made of synthetic material was placed | Postoperative anti-TB | 12 | 3 | No complications |
| [42] | 2009 | M 30 | TB | Pulmonary TB | Not mentioned | Ascending aorta | Rupture | Cardiac tamponade | Not mentioned | Open surgery: repair using Dacron graft with prosthetic aortic valve sewn into one end | Postoperative anti-TB | Not mentioned | 0.7 | Became afebrile |
| [43] | 2009 | M 69 | TB | Not sure | Smoking | DTA | Rupture | Shock | Yes | EVAR with Valiant thoracic  Stent graft (Medtronic Inc., 36 mm in diameter and 100 mm in length) | Postoperative anti-TB | Not mentioned | 4 days | No complications |
| [44] | 2010 | M 68 | TB | Pulmonary TB; lymphadenopathy TB | DM | Infrarenal AAA | Rupture | Hematemesis | Yes | Open surgery: aneurysm repaired with bifurcation Gortex graft, the distal limbs anastomosed to the common iliac arteries | Preoperative anti-TB | Not mentioned | Not mentioned | No complications |
| [45] | 2010 | F 38 | TB | TB of cervical lymph node | No | AAA (near the coeliac artery) | Rupture | Nothing specific | Not mentioned | EVAR with stent graft consisting of Ni-Ti Z-segments covered with eptfe (microport Life- sciences Co, Shanghai, China) | Preoperative anti-TB | Not mentioned | 18 | No complications |
| [46] | 2010 | 49 | TB | Miliary TB | Not mentioned | AAA (supraceliac (<10 mm from celiac artery origin)) | No rupture | Stable | No | EVAR with a right femoral arteriotomy to accommodate the custom-made Cook device (Cook 22/50; Cook Medical Inc, Bloomington, Ind) under epidural anesthesia | Preoperative anti-TB | 3 | 48 | No complications |
| [47] | 2010 | F 37 | TB | Military pulmonary TB; surgical specimen | No | DTA | Rupture | Nothing specific | Not mentioned | Open surgery: the affected aorta was debrided, and a true Dacron graft was inserted into the DTA | Preoperative anti-TB | Not mentioned | 8 | No complications |
| [48] | 2011 | M 59 | TB | Miliary involvement of the peritoneum | Not mentioned | Infrarenal AAA and thoracic aorta | No rupture | Nothing specific | No | Open surgery: aneurysm resected and repaired with a prosthetic graft | Preoperative anti-TB | 9 | 36 | 1 year later, the aneurysm increased and replaced with a Dacron tube graft; |
| [49] | 2011 | F 54 | TB | Miliary TB | Malaria, oophorectomy | DTA | No rupture | Nothing specific | No | EVAR with a Gore TAG endoprosthesis (W.L. Gore and associates, Flagstaff, AZ) | Preoperative anti-TB | 3 | 16 | No complications |
| [50] | 2011 | F 19 | TB | Lymphadenopathy TB | Not mentioned | Thoracic and abdominal aorta | No rupture | Nothing specific | No | Open surgery: aneurysm resected and replaced with patches of cryopreserved aortic allograft | Preoperative anti-TB | 9 | 9 | No complications |
| [51] | 2011 | M 75 | BCG | Military TB | Hypertension, dyslipidaemia, CKD, non-Hodgkin’s lymphoma, bladder cancer | Suprarenal AAA | No | Stable | No | Open surgery: an axillo bi-femoral graft was inserted and thoraco-laparotomy performed to explore the aneurysm | Preoperative anti-TB | Till death | 1 month | Died of aortic aneurysm rupture one month later |
| [52] | 2011 | F 32 | TB | Pulmonary TB | No | Juxtarenal AAA | Rupture | Anemia | Yes | Open surgery: resection of the aneurysm with repair of the aortic wall defect by a Dacron Silver patch. | Preoperative anti-TB | Not mentioned | 7 days | No complications |
| [53] | 2011 | M 39 | TB | Pulmonary TB | No | Infrarenal AAA | No | No | No | EVAR with stent graft | Under anti-TB | Not mentioned | Not mentioned | Paraplegia due to ischemic myelopathy postoperatively |
| [54] | 2011 | M 31 | TB | Pulmonary TB | No | DTA | No | Stable | Yes | EVAR with stent graft | Under anti-TB | Not mentioned | 24 | No complications |
| [55] | 2011 | M 62 | BCG | Disseminated TB | Bladder cancer | Infrarenal AAA | Rupture | Stable | Yes | Open surgery: extra-anatomic bypass and to ligate the aorta by oversewing both the proximal end below the renal arteries and the distal end at the aortic bifurcation; An axillo-bifemoral bypass graft using an 8 mm ringed polytetrafluoroethylene graft was then placed | Postoperative anti-TB | 12 | 16 days | No complications |
| [56] | 2012 | M 51 | TB | Military TB | Not mentioned | Proximal DTA | Rupture | Aneurysm increased in size | Yes | Open surgery: distal arch and proximal DTA resected and replaced with artificial graft | Preoperative anti-TB | 6 | 12 | No complications |
| [57] | 2012 | M 28 | TB | Military TB | Not mentioned | DTA | Rupture | Intubation for massive hemoptysis | Not mentioned | EVAR with stent graft | Preoperative anti-TB | Not mentioned | 9 | No complications |
| [58] | 2012 | F 16 | TB | Aneurysm tissue | Not mentioned | Infrarenal AAA | Rupture | Resuscitation | Not sure | EVAR with Multilayer stent (Cardiatis SA, Isnes, Belgium) | Postoperative anti-TB | 12 | 66 | Thoracic aneurysms increased at 2th month; 4 years later: 4 aneurysms of the descending and suprarenal aorta occurred and EVAR performed |
| [59] | 2012 | M 62 | TB | Military TB; Pott’s Disease | No | DTA | Rupture | Potential for increased bleeding | Yes | EVAR with Valiant thoracic stent graft (Medtronic, Fridley, MN, USA), covering the orifice of the left subclavian artery | Preoperative anti-TB | 5 | 12 | No complications |
| [60] | 2012 | M 68 | BCG | Aneurysm | Bladder cancer | Infrarenal AAA | Rupture | Hypotension | Yes | Open surgery: ligation of aorta and axillary-bifemoral bypass | Postoperative anti-TB | 12 months | 12 | AKI require hemodialysis and progressed on to ESRF after surgery |
| [61] | 2013 | M 81 | BCG | Not found | Bladder carcinoma | AAA (infrarenal) | No rupture | Nothing specific | No | EVAR with Zenith stent (Cook Inc., Bloomington, IN) | 24 months postoperative anti-TB; | Not mentioned | 6 | AAA infection associated with iliopsoas abscess and another open surgery at 6th month |
| [62] | 2013 | M 69 | TB | Disseminated TB | Stroke | AAA (infrarenal) | No rupture | Nothing specific | Not mentioned | EVAR with stent graft | Postoperative anti-TB | Till death | 1 | Cardiac arrest and died 1month after surgery |
| [63] | 2013 | M 40 | TB | Active miliary TB | Smoke and drinking | Distal aortic arch | No rupture | Aneurysm increased in size to three times | Yes | Open surgery; aneurysm dissected, debrided, the opening site was closed with direct pledgeted sutures | Preoperative anti-TB | Not mentioned | 24 | No complications |
| [64] | 2013 | M 58 | BCG | BCG-osis spinal TB (T8-9) | Myocardial infarction, stroke and prostatectomy bladder carcinoma; | AAA | No rupture | Not mentioned | No | EVAR | 14 months postoperative anti-TB | 12 | 30 | 6 months later, psoas abscess around the aorta persisted, and the graft removed |
| [65] | 2013 | M 63 | TB | Pulmonary TB | No | DTA | No rupture | Hemoptysis | Yes | EVAR with Gore-PTEF stent graft (TAG 3110), through the left femoral artery | Postoperative anti-TB | 4 | 12 | No complications |
| [66] | 2013 | M 69 | BCG | TB of aneurysm and the surround tissue | Bladder cancer, hypertension, hyperlipidemia, COPD | Infrarenal AAA | No rupture | Nothing specific | No | Open surgery: debridement of abscess cavity, reconstruction with cryopreserved aortic interposition graft | Preoperative anti-TB | 9 | 9 | No complications |
| [67] | 2013 | M 77 | BCG | Aneurysm | Smoke; Bladder cancer | Infrarenal AAA | Rupture | Stable | Yes | Open surgery: Surgical repair was performed using a 14 mm Gelsoft graft pre-soaked in Rifampycin | Postoperative anti-TB | Till death | 13 days | Perforation or anastomotic leak after 7 days and another surgery performed; AKI need hemofiltration; died 13 days later |
| [68] | 2014 | M 44 | TB | TB of kidney and vertebrae | Smoke | Infrarenal AAA | No rupture | Nothing specific | No | EVAR with a straight endograft+ a bare stent implanted to seal endoleaks after endograft stenting. | Preoperative anti-TB | 12 | 6 | No complications |
| [69] | 2014 | M 64 | BCG | Aneurysm | Gastroesophageal reflux disease, asthma, smoke, Bladder cancer | Suprarenal AAA | Rupture | Stable | No | Open surgery: debridement and repair with a 24-mm rifampin-soaked, four-branched, Coselli vascular graft | Preoperative anti-TB | 9 | 10 | No complications |
| [70] | 2014 | M 18 | TB | Pulmonary TB; spinal TB | No | Dissecting aortic root aneurysm | No | Stable | No | Open surgery: Bentall procedure was done with replacement of incompetent aortic valve and dilated ascending aorta with valved conduit of 25 mm mechanical valve size | Under anti-TB | Till death | 10 days | Died from ARDS 10 days after surgery due to ARDS |
| [71] | 2015 | F 40 | TB | Tuberculous aortitis | No | Ascending aorta | No rupture | Nothing specific | No | Open surgery: aortic valve replacement and aortic root replacement | Postoperative anti-TB | 10 | 10 | No complications |
| [72] | 2015 | F 31 | TB | Disseminated TB; pulmonary and brain TB | No | Ascending aorta | Rupture during surgery | Nothing specific | No | Open surgery: Repair with ascending aorta patch collage coated polyester and pericardiectomy | Preoperative anti-TB | 9 | 12 | No complications |
| [73] | 2015 | M 81 | BCG | BCG-osis (granulomatous hepatitis) | Smoke, COPD, bladder carcinoma; granulomatous hepatitis | Aortic arch, | No rupture | Nothing specific | No | Open surgery: resection of aneurysm, proximal left subclavian artery and regional arch, reconstruction with a homograft patch | Preoperative anti-TB | 12 | 12 | No complications |
| [74] | 2015 | M 59 | TB | Spinal TB (T6) | No | DTA | No rupture | Nothing specific | No | EVAR with a covered stent (Medtronic TF 3030C200EE) | Preoperative anti-TB | Not mentioned | 14 | No complications |
| [75] | 2015 | M 64 | BCG | BCG-osis | Bladder carcinoma; hypertension, hyperlipidemia, stroke, smoke | Thoracoabdominal aorta | Rupture | Nothing specific | No | Open surgery: debridement of distal aorta, proximal aortic end-to-side anastomosis, sequential visceral reimplantation with rifampin-soaked Dacron graft , followed by distal aortic end-to-end anastomosis and interval ligation of the aorta | 27 days postoperative anti-TB | 9 | 11 | No complications |
| [76] | 2015 | M 28 | TB | Spinal tuberculous | Not mentioned | DTA | No | Stable | No | EVAR with endoprosthesis | Anti-TB | 6 | 24 | No complications |
| [77] | 2015 | M 70 | BCG | Aneurysm | Bladder cancer | AAA | No | Stable | No | Open surgery: excision of the aortic aneurysm and  Extensive debridment of the periaortic tissue and aortic reconstruction with a 14-mm Dacron graft | Postoperative anti-TB | Not mentioned | 3 | No complications |
| [78] | 2015 | M 23 | TB | Aneurysm | Prolactinoma,2 | Aneurysms in  Aortic isthmus, origin of the brachiocephalic artery trunk, origin of the left CCA | No | Stable | No | Open surgery: a bypass surgery with placement of a Y Dacron tube from the ascendant aorta to the right brachiocephalic trunk and left common carotid, implantation of an endoprosthesis in the ascending aorta subsequent to the bypass towards the DTA with aneurysm exclusion | Preoperative anti-TB | 6 | Not mentioned | No complications |
| [79] | 2016 | M 18 | TB | Pulmonary TB | No | Aneurysms from the distal aortic arch up to the aortic bificuration | No rupture | Noting specific | Not specify | Open surgery: the distal arch was reconstructed; the left renal artery was anastomosed using an interposition polyethylene terephthalate graft. | Preoperative anti-TB | 6 | 6 | No complications |
| [80] | 2016 | M 73 | TB | Aneurysm | Smoking, ischemic cardiopathy and hypertension | Pararenal AAA | No | Stable | No | Open surgery: Under suprarenal clamping, the aneurysm was partially resected and, usinga bifurcated Dacron graft, an aorto-bifemoral interpositionand a bypass to the left renal artery done | Postoperative anti-TB | Not mentioned | 8 | No complications |
| [81] | 2017 | F 77 | TB | Chest wound TB; pulmonary TB; | Schizophrenia; nonhealing wound on the chest wall | Ascending aorta | Rupture | Massive hemoptysis; and sudden hypotension | No sure | TEVAR with stent graft (Valiant Thoracic Stent Graft with the Captivia Delivery System) under general anesthesia | Postoperative anti-TB | 9 | 12 | No complications |
| [82] | 2017 | M 84 | TB | Miliary TB | Smoke | AAA at the level of the left renal artery | No rupture | Noting specific | No | EVAR with intraluminal stent | 1.5 months postoperative anti-TB | 9 | 9 | No complications |
| [83] | 2017 | M 72 | BCG | Aneurysm | Bladder cancer | Infrarenal AAA | No | Stable | No | Open surgery: resection of his aneurysms with inline reconstruction utilizing a rifampin-soaked Gelsoft bifurcated graft | Postoperative anti-TB | 6 | 1 | Chylothorax and GI bleed requiring after surgery |
| [84] | 2017 | M 79 | TB | Spinal TB | No | Infrarenal AAA | Rupture | Stable | Yes | EVAR with stent graft using Endurant II AAA Stent Graft System | Postoperative anti-TB | 12 | Not mentioned | Recovered well |
| [85] | 2018 | M 66 | BCG | TB in childhood BCG-osis | Bladder cancer, hypertension dyslipidemia CAD, smoke | Infrarenal AAA | Rupture | Nothing specific | Not specify | Open surgery: surgical flattening of the aneurysm | Postoperative anti-TB | 10 | 9 | No complications |
| [86] | 2018 | M 76 | BCG | Pott disease (L2/3) | Bladder cancer hypertension, DM, idiopathic skeletal hyperostosis | AAA | No rupture | Nothing specific | No | Open surgery: replace the aneurysm with a synthetic graft | Postoperative anti-TB | Not mentioned | 2.5 | Nothing specific |
| [87] | 2018 | F 24 | TB | Pulmonary TB; thoracic spinal TB with paraspinal abscess | Not mentioned | Thoracic aorta | Rupture | Not specify | Not specify | EVAR with stent graft | Postoperative anti-TB | Not mentioned | 18 | No complications |
| [88] | 2018 | M 65 | BCG | Pulmonary TB; | Bladder cancer | Thoracic aorta | Rupture | Hypovolemic shock | Not sure | TEVAR | 160 days postoperative anti-TB | Not mentioned | 6 | No complications |
| [89] | 2018 | M 26 | TB | Pulmonary TB | No | DTA | No | Stable | Yes | EVAR | Preoperative anti-TB | Not mentioned | 1 | No complications |
| [90] | 2018 | M 73 | BCG | Aneurysm | Bladder cancer | Pararenal AAA | No | Stable | No | Open surgery: infected aneurysmal segment was  Excised, reconstructive repair was performed with a bovine (pericardial) tube graft. | Postoperative anti-TB | Not mentioned | 5 months | Acute-on-chronic renal insufficiency developed after surgery and hemodialysis; 5 months later, abscess formation |
| [91] | 2018 | M 73 | BCG | Aneurysm | Type II diabetes, hypertension, dyslipidemia, and macular degeneration, bladder cancer | DTA | No | Stable | No | EVAR | Preoperative anti-TB | Not mentioned | 6 | No complications |
| [91] | 2018 | M 67 | BCG |  | Bladder cancer | Infrarenal AAA | No | Stable | No | Open surgery: surgical  Resection of the infected infrarenal aortic segment and repaired using an autologous graft harvested from the patient’s  Left femoral vein | Postoperative anti-TB | Not mentioned | Not mentioned | No complications |
| [92] | 2018 | M 75 | BCG | Multiple | Arterial hypertension, peripheral arterial disease, chronic renal failure | Infrarenal AAA | No | Stable | No | Open surgery: place an aorto-bifemoral vascular prosthesis | Delayed anti-TB | 6 | Not mentioned | No complications |
| [93] | 2019 | 66 F | TB | Pulmonary TB; spinal TB (T3/T4) | No | Aortic arch | Rupture | Hemorrhage and cardiac arrest | Yes | EVAR with stent-graft | Preoperative anti-TB | Not mentioned | 32 | No complications |
| [94] | 2019 | 79 M | BCG | Disseminated mycobacterial infection | Bladder cancer hypertension ischemic attack; smoke | Aortic arch | No rupture | Aneurysm increase within 5 months, | No | TEVAR with a single component Cook Thoracic Alpha stent- graft. | Postoperative anti-TB | Not mentioned | 1.4 | No complications |
| [95] | 2019 | M 70 | BCG |  | Hypertension, hyperlipidemia, ischemic heart disease, bladder cancer | Infrarenal AAA | Rupture | Stable | No | EVAR with stent (BA25-90/120-30 AFX;  Endologix Inc., Irvine, CA) through a bilateral groin cut down approach | Not mentioned | Not mentioned | 25 days | Aortoenteric fistula 10 days after surgery, the endograft was removed and axillobifemoral bypass was performed; critical colic ischemia requiring total colectomy. Died 25 days later. |
| [96] | 2019 | F 57 | TB | Pulmonary TB; Pott disease (T11/12) | No | Suprarenal AAA | Rupture | Unstable | Yes | EVAR by positioning an Ankura thoracic stent graft (28mm at diameter; 160mm at length; Lifetech, Shenzhen, China) | Preoperative anti-TB | 12 | 24 | No complications |
| [97] | 2020 | F 42 | TB | Disseminated TB (bilateral lung, spinal TB with paravertebral abscess) | No | DTA | Rupture | Stable | Emergency | The aneurysm ruptured 16 months after anti-TB; TEVAR performed with Valiant Thoracic stent-graft (Medtronic Vascular, Santa Rosa, CA, USA) | Preoperative anti-TB | 14 | 9 | Stent graft infection at 7th; died at 9 th month from shock |
| [98] | 2020 | M 47 | TB | Pulmonary TB | Hypertension; alcohol consumption | Ascending aorta | Rupture | Massive hemoptysis; para-aortic mass increased | Yes | Open surgery: hemi-arch replacement with vascular prosthesis | Preoperative anti-TB | 12 | 10 | No complications |
| [99] | 2020 | M 15 | TB | Cervical nodes TB | Takayasu arteritis | Ascending aorta | No rupture | Stable | No | Open surgery: ascending aorta and total aortic arch replacement | Preoperative anti-TB | Not mentioned | 6 | No complications |
| [100] | 2020 | F 26 | TB | Pulmonary TB | HIV | Proximal descending aorta, | Rupture | Unstable | No | EVAR and lung resection | Postoperative anti-TB | 9 | 12 | No complications |
| [101] | 2020 | M 30 | TB | Pulmonary TB | No | DTA | Rupture | Massive hemoptysis | Yes | EVAR with thoracic stent-graft (Valiant Captivia  Thoracic Stent, Medtronic Inc., Minneapolis, MN, USA) | Under anti-TB | Till death | 4 | Died of respiratory failure 4 months later |
| [101] | 2020 | F 26 | TB | Brain | No | DTA | Rupture | Massive hemoptysis | Yes | EVAR with thoracic stent-graft (Valiant Captivia Thoracic Stent, Medtronic Inc.) | Under anti-TB | 9 | 60 | No complications |
| [102] | 2021 | F 75 | TB | Multiple TB | No | DTA | No | Stable | No | EVAR with endoprothesis (Cook Zenith alpha) | Under anti-TB | 11 | 3 | No complications |
| [103] | 2021 | M 63 | BCG | Aneurysm | Bladder cancer | Infrarenal AAA | Rupture | Unstable | Yes | EVAR with an aortobiiliac graft | 24 months after surgery | 12 | 84 | No complications |
| [103] | 2021 | M 78 | BCG | Spinal TB | Bladder cancer | Infrarenal AAA | Rupture | Stable | No | Open surgery: aortic reconstruction was performed  By implantation of a rifampicin-soaked Dacron tube graft | 4 months after surgery | 30 | 35 | 23 months later, a false aneurysm near the proximal anastomosis of the Dacron tube graft, and Graft explantation and replacement with homograft was performed |
| [103] | 2021 | M 79 | BCG | Aneurysm | Bladder cancer | Infrarenal AAA | Rupture | Stable | No | Open surgery: Implantation of a Dacron tube graft | Postoperative anti-TB | Not mentioned | 18 | 6 months later, a false aneurysm in the infrarenal region near the proximal anastomosis of the Dacron tube graft, the Dacron tube graft was removed and replaced by deep femoral vein |
| [104] | 2021 | M 35 | TB | Aneurysm | TB | Thoraco-abdominal aorta | No | Stable | No | Open surgery: iliac-mesenteric and iliac femoral bypasses were put in place and a covered aortouniliac endoprosthesis was inserted. | Postoperative anti-TB | 10 months | 6 months | No complications |
| [105] | 2021 | M 80 | BCG | Aneurysm | Bladder cancer | DTA | No | Stable | No | EVAR | Under anti-TB | Not mentioned | 24 | No complications |
| [106] | 2021 | M 35 | TB | Pulmonary and lumbar t TB | No | Infrarenal AAA | Rupture | Stable | Yes | EVAR | Under anti-TB | Not mentioned | 10 | No complications |
| [106] | 2021 | M 23 | TB | Not mentioned |  | Thoracoabdominal aorta | Rupture | Stable | Yes | EVAR | Under anti-TB | Not mentioned | 16 | Paralytic ileus 3 days later |
| [107] | 2021 | F 45 | TB | Disseminated TB | No | Aortic arch | Rupture | Unstable | Yes | EVAR | Preoperative anti-TB | 18 months | 18 | No complications |

TB: tuberculosis; BCG: Bacillus Calmette-Guerin; EVAR: endovascular aneurysm repair; CAD: chronic coronary disease; COPD: chronic obstructive pulmonary disease; Pott disease: tuberculous spondylitis; DTA: descending thoracic aorta; PTFE: polytetrafluorethylene; ePTFE: expanded polytetrafluoroethylene; AAA: abdominal aortic aneurysm;

**Reference**

1. Ohtsuka T, Kotsuka Y, Yagyu K, Furuse A, Oka T. Tuberculous pseudoaneurysm of the thoracic aorta. Annals of Thoracic Surgery. 1996;62(6):1831-4.

2. Ikezawa T, Iwatsuka Y, Naiki K, Asano M, Ikeda S, Kimura A. Tuberculous pseudoaneurysm of the descending thoracic aorta: A case report and literature review of surgically treated cases. Journal of Vascular Surgery. 1996;24(4):693-7.

3. Hagino RT, Clagett GP, Valentine RJ. A case of Pott's disease of the spine eroding into the suprarenal aorta. J Vasc Surg. 1996;24(3):482-6.

4. Rozenblit A, Wasserman E, Marin ML, Veith FJ, Cynamon J, Rozenblit G. Infected aortic aneurysm and vertebral osteomyelitis after intravesical bacillus Calmette-Guérin therapy. AJR Am J Roentgenol. 1996;167(3):711-3.

5. Bojar RM, Turner MT, Valdez S, Haskal R, McGowan K, Khabbaz KR. Homograft repair of a tuberculous pseudoaneurysm of the ascending aorta. Chest. 1998;114(6):1774-6.

6. Damm O, Briheim G, Hagström T, Jönsson B, Skau T. Ruptured mycotic aneurysm of the abdominal aorta: a serious complication of intravesical instillation bacillus Calmette-Guerin therapy. J Urol. 1998;159(3):984.

7. Golzarian J, Cheng J, Giron F, Bilfinger TV. Tuberculous pseudoaneurysm of the descending thoracic aorta: Successful treatment by surgical excision and primary repair. Texas Heart Institute Journal. 1999;26(3):232-5.

8. Baltacioğlu F, Cimşit NC, Aribal ME. Tuberculous abdominal aortic aneurysm in a 14-year-old child. Pediatr Radiol. 1999;29(7):536-8.

9. Allins AD, Wagner WH, Cossman DV, Gold RN, Hiatt JR. Tuberculous infection of the descending thoracic and abdominal aorta: case report and literature review. Ann Vasc Surg. 1999;13(4):439-44.

10. Seelig MH, Oldenburg WA, Klingler PJ, Blute ML, Pairolero PC. Mycotic vascular infections of large arteries with Mycobacterium bovis after intravesical bacillus Calmette-Guérin therapy: case report. J Vasc Surg. 1999;29(2):377-81.

11. Long R, Guzman R, Greenberg H, Safneck J, Hershfield E. Tuberculous mycotic aneurysm of the aorta: review of published medical and surgical experience. Chest. 1999;115(2):522-31.

12. Liu WC, Kwak BK, Kim KN, Kim SY, Woo JJ, Chung DJ, et al. Tuberculous aneurysm of the abdominal aorta: endovascular repair using stent grafts in two cases. Korean journal of radiology : official journal of the Korean Radiological Society. 2000;1(4):215-8.

13. de Kruijf EJ, van Rijn AB, Koelma IA, Kuijpers TJ, van 't Wout JW. Tuberculous aortitis with an aortoduodenal fistula presenting as recurrent gastrointestinal bleeding. Clin Infect Dis. 2000;31(3):841-2.

14. Akgun S, Civelek A, Akalin F. Mycotic aneurysm of suprarenal aorta in a 14-year-old boy. Asian Cardiovascular and Thoracic Annals. 2000;8(2):169-71.

15. Strnad BT, McGraw JK, Heatwole EV, Clark P. Tuberculous aneurysm of the aorta presenting with uncontrolled hypertension. Journal of Vascular and Interventional Radiology. 2001;12(4):521-3.

16. Choudhary SK, Bhan A, Talwar S, Goyal M, Sharma S, Venugopal P. Tubercular pseudoaneurysms of aorta. Ann Thorac Surg. 2001;72(4):1239-44.

17. Kamphuis JT, Buiting AG, Miseré JF, van Berge Henegouwen DP, van Soolingen D, Rensma PL. BCG immunotherapy: be cautious of granulomas. Disseminated BCG infection and mycotic aneurysm as late complications of intravesical BCG instillations. Neth J Med. 2001;58(2):71-5.

18. Hatem CM, Kantis GA, Christoforou D, Gold JP, Plestis KA. Tuberculous aneurysm of the descending thoracic aorta. Journal of Thoracic and Cardiovascular Surgery. 2002;123(2):373-4.

19. Shigemitsu O, Hadama T, Miyamoto S, Anai H, Sako H. Tuberculous pseudoaneurysm of the ascending aorta associated with intracranial tuberculoma. Journal of Cardiovascular Surgery. 2002;43(1):59-62.

20. Suresh K, Kurian V, Madhu Sankar N, Patel A, Joseph P, Cherian KM. Repair of tuberculous aneurysm of distal aortic arch. Asian Cardiovascular and Thoracic Annals. 2003;11(4):346-8.

21. Abad C, Santamaria P. Tuberculous aneurysm of the aortic arch. J Thorac Cardiovasc Surg. 2003;126(4):1229.

22. Choi JB, Yang HW, Oh SK, Yun KJ. Rupture of ascending aorta secondary to tuberculous aortitis. Ann Thorac Surg. 2003;75(6):1965-7.

23. Aebert H, Birnbaum DE. Tuberculous pseudoaneurysms of the aortic arch. Journal of Thoracic and Cardiovascular Surgery. 2003;125(2):411-2.

24. Lee EB, Lee SC, Cho JY, Lee JT. Surgery for concomitant aortoesophageal and aortobronchial fistula in tuberculous aortitis. Interact Cardiovasc Thorac Surg. 2003;2(3):234-6.

25. Forbes TL, Harris JR, Nie RG, Lawlor DK. Tuberculous aneurysm of the supraceliac aorta--a case report. Vasc Endovascular Surg. 2004;38(1):93-7.

26. Falkensammer J, Behensky H, Gruber H, Prodinger WM, Fraedrich G. Successful treatment of a tuberculous vertebral osteomyelitis eroding the thoracoabdominal aorta: a case report. J Vasc Surg. 2005;42(5):1010-3.

27. Shikata H, Nagayoshi Y, Takeuchi K, Ueda Y, Sakamoto S, Kanno M, et al. Successful surgical treatment of an infrarenal abdominal pseudoaneurysm caused by tuberculosis: Report of a case. Surgery Today. 2005;35(11):991-5.

28. Dahl T, Lange C, Ødegård A, Bergh K, Osen SS, Myhre HO. Ruptured abdominal aortic aneurysm secondary to tuberculous spondylitis. Int Angiol. 2005;24(1):98-101.

29. Bukhary ZA, Alrajhi AA. Tuberculous aortitis. Ann Saudi Med. 2006;26(1):56-8.

30. Chen SH, Wong T, Kuo FY, Lee CH. Tuberculous spondylitis and salmonella mycotic aneurysm in an immunocompromised patient. A case report. J Bone Joint Surg Am. 2006;88(10):2275-8.

31. Labrousse L, Montaudon M, Le Guyader A, Choukroun E, Laurent F, Deville C. Endovascular treatment of a tuberculous infected aneurysm of the descending thoracic aorta: a word of caution. J Vasc Surg. 2007;46(4):786-8.

32. Loh YJ, Tay KH, Mathew S, Tan KL, Cheah FK, Sin YK. Endovascular stent graft treatment of leaking thoracic aortic tuberculous pseudoaneurysm. Singapore Medical Journal. 2007;48(7):e193-e5.

33. Harding GE, Lawlor DK. Ruptured mycotic abdominal aortic aneurysm secondary to Mycobacterium bovis after intravesical treatment with bacillus Calmette-Guérin. J Vasc Surg. 2007;46(1):131-4.

34. Jain AK, Chauhan RS, Dhammi IK, Maheshwari AV, Ray R. Tubercular pseudoaneurysm of aorta: a rare association with vertebral tuberculosis. Spine J. 2007;7(2):249-53.

35. Tsai HC, Lee SSJ, Wann SR, Chen YS, Wang JS, Chen ER, et al. Recurrent gastrointestinal bleeding due to a tuberculous mycotic aneurysm with an aortoduodenal fistula. International Journal of Infectious Diseases. 2007;11(2):182-4.

36. Takahashi Y, Sasaki Y, Shibata T, Suehiro S. Descending thoracic aortic aneurysm complicated with severe vertebral erosion. Eur J Cardiothorac Surg. 2007;31(5):941-3.

37. Yotsumoto H. Fever, hypercalcemia and tuberculosis. Intern Med. 2007;46(6):259-60.

38. Canaud L, Marzelle J, Bassinet L, Carrié AS, Desgranges P, Becquemin JP. Tuberculous aneurysms of the abdominal aorta. J Vasc Surg. 2008;48(4):1012-6.

39. Hussein H, Azizi ZA. Tuberculous aortic pseudoaneurysm treated with in situ silver-impregnated vascular inlay graft. Asian Journal of Surgery. 2008;31(2):87-9.

40. Tsai TJ, Yu HC, Lai KH, Lo GH, Hsu PI, Fu TY. Primary aortoduodenal fistula caused by tuberculous aortitis presenting as recurrent massive gastrointestinal bleeding. J Formos Med Assoc. 2008;107(1):77-83.

41. Safdar N, Abad CL, Kaul DR, Jarrard D, Saint S. Clinical problem-solving. An unintended consequence--a 79-year-old man with a 5-month history of fatigue and 20-lb (9-kg) weight loss presented to his local physician. N Engl J Med. 2008;358(14):1496-501.

42. Palaniswamy C, Kumar U, Selvaraj DR, Pandey B, Handa R, Alappan NK, et al. Tuberculous mycotic aneurysm of aortic root: an unusual cause of cardiac tamponade. Trop Doct. 2009;39(2):112-3.

43. Dogan S, Memis A, Kale A, Buket S. Endovascular stent graft placement in the treatment of ruptured tuberculous pseudoaneurysm of the descending thoracic aorta: Case report and review of the literature. CardioVascular and Interventional Radiology. 2009;32(3):572-6.

44. Chong VH, Telisinghe PU, Chong CF. Tuberculous aorto-duodenal fistula: a rare cause of upper gastrointestinal bleeding. Singapore Med J. 2010;51(5):e85-8.

45. Shu C, He H, Li QM, Li M, Jiang XH, Li X. Endovascular percutaneous treatment of tuberculous pseudo-aneurysm involving the coeliac artery: a case report. Eur J Vasc Endovasc Surg. 2010;40(2):230-3.

46. Clough RE, Topple JA, Zayed HA, Lyons OT, Carrell TW, Taylor PR. Endovascular repair of a tuberculous mycotic thoracic aortic aneurysm with a custom-made device. J Vasc Surg. 2010;51(5):1272-5.

47. Park SC, Moon IS, Koh YB. Tuberculous Pseudoaneurysm of the Descending Thoracic Aorta. Annals of Vascular Surgery. 2010;24(3):e11-417.

48. Avaro JP, Amabile P, Paule P, Peloni JM, Piquet P. An unusual combination of a tuberculous aneurysm of the thoracic aorta and a degenerative aneurysm of the infrarenal abdominal aorta. Annals of Vascular Surgery. 2011;25(5):e9-700.

49. Han DK, Chung C, Walkup MH, Faries PL, Marin ML, Ellozy SH. Endovascular stent-graft repair of a tuberculous mycotic aortic aneurysm. Ann Vasc Surg. 2011;25(5):699.e13-6.

50. Pierret C, Tourtier JP, Grand B, Boddaert G, Laurian C, De Kerangal X. Multiple tuberculous aneurysms of the aorta. Journal of Vascular Surgery. 2011;53(6):1720-2.

51. Maundrell J, Fletcher S, Roberts P, Stein A, Lambie M. Mycotic aneurysm of the aorta as a complication of Bacillus Calmette-Guérin instillation. J R Coll Physicians Edinb. 2011;41(2):114-6.

52. Mechchat A, Lekehal B, Mesnaoui A, Ammar F, Bensaid Y. Ruptured tuberculous false aneurysm of the abdominal aorta: a case report. Ann Vasc Dis. 2011;4(1):47-9.

53. Rekha P, Sukumaran P, Jayakumar TK, Venugopal K. Tuberculous mycotic aneurysm of aorta: A rare complication. European Respiratory Journal. 2011;38.

54. Wang Y, Zhang J, Yin MD, Wang SY, Duan ZQ, Xin SJ. Endovascular repair of a tuberculous aneurysm of descending thoracic aorta. Chinese Medical Journal. 2011;124(14):2228-30.

55. Pittman M, Sakai L, Craig R, Joehl R, Milner R. Primary aortoenteric fistula following disseminated bacillus Calmette-Guérin infection: a case report. Vascular. 2012;20(4):221-4.

56. Seo DJ, Kim JB. Tuberculous aortitis complicated with pseudoaneurysm formation in the descending thoracic aorta: a case report. Korean J Thorac Cardiovasc Surg. 2012;45(6):408-11.

57. Koh J, Law J, Raghuram J. Massive hemoptysis, not your usual tuberculosis. Am J Respir Crit Care Med. 2012;185(4):455.

58. Benjelloun A, Henry M, Ghannam A, Vaislic C, Azzouzi A, Maazouzi W, et al. Endovascular treatment of a tuberculous thoracoabdominal aneurysm with the multilayer stent. Journal of Endovascular Therapy. 2012;19(1):115-20.

59. Li FP, Wang XF, Xiao YB. Endovascular stent graft placement in the treatment of a ruptured tuberculous pseudoaneurysm of the descending thoracic aorta secondary to Pott's disease of the spine. J Card Surg. 2012;27(1):75-7.

60. Khandelwal A, Gupta A, Virmani V, Khandelwal K. Ruptured Aortic Aneurysm Secondary to Psoas abscess after Intravesical Bacilli Calmette-Guérin. Med J Malaysia. 2012;67(5):534-5.

61. Mizoguchi H, Iida O, Dohi T, Tomoda K, Kimura H, Inoue K, et al. Abdominal aortic aneurysmal and endovascular device infection with iliopsoas abscess caused by Mycobacterium bovis as a complication of intravesical bacillus Calmette-Guérin therapy. Ann Vasc Surg. 2013;27(8):1186.e1-5.

62. Mearelli F, Burekovic I, Zanetti M, Altamura N, Carlo G, Biolo G, et al. Disseminated tuberculosis in an immunocompetent patient. International Journal of Infectious Diseases. 2013;17(9):e784-e6.

63. Cho WC, Yoo DG, Kim JW, Park CB. Primary repair of tuberculous pseudoaneurysm of the aortic arch. Journal of Cardiac Surgery. 2013;28(5):563-6.

64. Santbergen B, Vriens PH, de Lange WC, Van Kasteren ME. Combined infection of vertebroplasty and aortic graft after intravesical BCG treatment. BMJ Case Rep. 2013;2013.

65. Marjanovic I, Sarac M, Tomic A, Bezmarevic M. Endovascular repair of mycotic aneurysm of the descending thoracic aorta: diagnostic and therapeutic dilemmas-two case reports with 1-year follow-up. Thorac Cardiovasc Surg. 2013;61(7):597-9.

66. Psoinos CM, Simons JP, Baril DT, Robinson WP, Schanzer A. A Mycobacterium bovis mycotic abdominal aortic aneurysm resulting from bladder cancer treatment, resection, and reconstruction with a cryopreserved aortic graft. Vasc Endovascular Surg. 2013;47(1):61-4.

67. Roylance A, Mosley J, Jameel M, Sylvan A, Walker V. Aorto-enteric fistula development secondary to mycotic abdominal aortic aneurysm following intravesical bacillus Calmette-Guerin (BCG) treatment for transitional cell carcinoma of the bladder. Int J Surg Case Rep. 2013;4(1):88-90.

68. Zhang C, Chen B, Gu Y, Luo T, Yang S, Liang W, et al. Tuberculous abdominal aortic pseudoaneurysm with renal and vertebral tuberculosis: A case and literature review. Journal of Infection in Developing Countries. 2014;8(9):1216-21.

69. Holmes BJ, LaRue RW, Black JH, 3rd, Dionne K, Parrish NM, Melia MT. Mycotic aortic aneurysm due to intravesical BCG immunotherapy: Clinical manifestations and diagnostic challenges. Int J Mycobacteriol. 2014;3(1):60-5.

70. Sharma D, Goyal G, Sisodia A, Devgarha S, Mathur RM. Dissecting aortic root aneurysm and severe aortic regurgitation following pulmonary tuberculosis. Egyptian Journal of Chest Diseases and Tuberculosis. 2014;63(2):523-7.

71. Pathirana U, Kularatne S, Karunaratne S, Ranasinghe G, Fernando J. Ascending aortic aneurysm caused by Mycobacterium tuberculosis. BMC Res Notes. 2015;8:659.

72. Gopalan S, Ramadurai S, Nair AM, Arthur P. Tuberculous pseudoaneurysm of the ascending aorta. BMJ Case Reports. 2015;2015 (no pagination)(211910).

73. Ventosa-Fernandez G, Milisenda JC, Pereda D, Mestres CA. Infected False Aneurysm of the Aortic Arch After Endoscopic Transurethral Instillation of Bacillus Calmette-Guérin. Ann Thorac Surg. 2015;100(2):717-20.

74. Zhao JC. Interventional Therapy for Infective Pseudoaneurysm is a Hope for a Patient in an Undeveloped Area. Arch Iran Med. 2015;18(6):389-90.

75. Davis FM, Miller DJ, Newton D, Arya S, Escobar GA. Successful treatment of a mycotic multifocal thoracoabdominal aortic aneurysm as a late sequelae of intravesical bacillus Calmette-Guerin therapy: case report and literature review. Ann Vasc Surg. 2015;29(4):840.e9-13.

76. Gualis J, Castano M, Gomez-Plana J, Fernandez-Vazquez F. Endovascular treatment of a tuberculous aneurysm of the descending thoracic aorta in a young patient. European Journal of Cardio-thoracic Surgery. 2015;48(2):336.

77. Leo E, Molinari AL, Rossi G, Ferrari SA, Terzi A, Lorenzi G. Mycotic abdominal aortic aneurysm after adjuvant therapy with bacillus Calmette-Guérin in patients with urothelial bladder cancer: a rare but misinterpreted complication. Ann Vasc Surg. 2015;29(6):1318.e1-6.

78. Moura C, Aquino MA, Rocha Filho J, Santiago M. Takayasu's or tuberculous arteritis? BMJ Case Rep. 2015;2015.

79. Velayudhan BV, Idhrees AM, Sahu D, Jacob A. Open surgical repair of multiple tuberculous mycotic aneurysms of the thoracoabdominal aorta. J Thorac Cardiovasc Surg. 2016;152(4):e95-8.

80. Manuel V, Tiago J, Martins P, Martins C, Nunes JS, Fernandes e Fernandes J. Tuberculous aortitis, a case report. Angiologia e Cirurgia Vascular. 2016;12(4):283-6.

81. Chiang WC, Ling-Lin Pai E, Chen PL, Shih CC, Chen IM. Endografting under Assistance of Transapical Body Floss Through-and-Through Wiring Technique and Rapid Ventricular Pacing for an Ascending Aortic Tuberculous Pseudoaneurysm. Annals of Vascular Surgery. 2017;45:e7-270.

82. Manika K, Efthymiou C, Damianidis G, Zioga E, Papadaki E, Lagoudi K, et al. Miliary tuberculosis in a patient with tuberculous mycotic aneurysm of the abdominal aorta: Case report and review of the literature. Respiratory Medicine Case Reports. 2017;21:30-5.

83. Coddington ND, Sandberg JK, Yang C, Sehn JK, Kim EH, Strope SA. Mycotic Aneurysm after Bacillus Calmette-Guérin Treatment: Case Report and Review of the Literature. Case Rep Urol. 2017;2017:4508583.

84. Pluemvitayaporn T, Jindahra S, Pongpinyopap W, Kunakornsawat S, Thiranon C, Singhatanadgige W, et al. Concomitant mycotic abdominal aortic aneurysm and lumbartuberculous spondylitis with cauda equina syndrome: a rare condition - a case report and literature review. Spinal Cord Ser Cases. 2018;4:13.

85. Darriet F, Bernioles P, Loukil A, Saidani N, Eldin C, Drancourt M. Fluorescence in situ hybridization microscopic detection of Bacilli Calmette Guérin mycobacteria in aortic lesions: A case report. Medicine (Baltimore). 2018;97(30):e11321.

86. Kusakabe T, Endo K, Nakamura I, Suzuki H, Nishimura H, Fukushima S, et al. Bacille Calmette-Guérin (BCG) spondylitis with adjacent mycotic aortic aneurysm after intravesical BCG therapy: a case report and literature review. BMC Infect Dis. 2018;18(1):290.

87. Xue J, Yao Y, Liu L. Treatment of tuberculous aortic pseudoaneurysm associated with vertebral tuberculosis. Medicine (United States). 2018;97(15) (no pagination)(e0382).

88. Higashi Y, Nakamura S, Kidani K, Matumoto K, Kawago K, Isobe J, et al. Mycobacterium bovis-induced Aneurysm after Intravesical Bacillus Calmette-Guérin Therapy: A Case Study and Literature Review. Intern Med. 2018;57(3):429-35.

89. Ikeda S, Youdelman BA, Shih M, Rhee RY. Case report of endovascular repair for tuberculous pseudoaneurysm in the descending aorta with computed tomographic-guided drainage of abscess and a literature review. Innovations: Technology and Techniques in Cardiothoracic and Vascular Surgery. 2018;13(Supplement 1):S22-S3.

90. Roeke T, Hovsibian S, Schlejen PM, Dinant S, Koster T, Waasdorp EJ. A mycotic aneurysm of the abdominal aorta caused by Mycobacterium bovis after intravesical instillation with bacillus Calmette-Guérin. J Vasc Surg Cases Innov Tech. 2018;4(2):122-5.

91. Wadhwani A, Moore RD, Bakshi D, Mirakhur A. Mycotic aortic aneurysms post-Intravesical BCG treatment for early-stage bladder carcinoma. CVIR Endovasc. 2018;1(1):28.

92. Lareyre F, Reverso-Meinietti J, Carboni J, Gaudart A, Hassen-Khodja R, Raffort JM. Mycotic Aortic Aneurysm and Infected Aortic Graft After Intravesical Bacillus Calmette-Guérin Treatment for Bladder Cancer. Vasc Endovascular Surg. 2019;53(1):86-91.

93. Tang H, Liu Y, Moro A, Xiao Z, Zhan X. Intraoperative rupture of tuberculous pseudoaneurysm associated with spinal tuberculosis: A case report and literature review. Journal of Infection in Developing Countries. 2019;13(2):174-8.

94. Ribeiro L, Rajendran S, Stenson K, Loftus I. Rare case of a proximal descending thoracic aorta mycotic aneurysm following intravesical BCG injections for the treatment of bladder cancer. BMJ Case Rep. 2019;12(12).

95. Berchiolli R, Mocellin DM, Marconi M, Tomei F, Bargellini I, Zanca R, et al. Ruptured Mycotic Aneurysm After Intravesical Instillation for Bladder Tumor. Ann Vasc Surg. 2019;59:310.e7-.e11.

96. Li W, Sun X, Li H, Meng Z, Yang Y, Yao S. Endovascular treatment of a ruptured thoracic aortic pseudoaneurysm secondary to Pott disease during a spine surgery : A case report and a literature review. Medicine (United States). 2019;98(16) (no pagination)(e15306).

97. Vijayvergiya R, Kasinadhuni G, Sinha SK, Yadav TD, Singh H, Savlania A, et al. Thoracic endovascular aortic repair in management of aorto-oesophageal fistulas: a case series. Eur Heart J Case Rep. 2020;4(5):1-6.

98. De Smet D, Payen MC, Remes J, Van den Wijngaert S, Vouche M, Konopnicki D. Tuberculosis and pseudoaneurysms. Med Mal Infect. 2020;50(5):446-50.

99. Mimbimi C, Hajj-Chahine J, Allain G, Jayle C, Corbi P. Dissecting Thoracic Aneurysm in Takayasu Arteritis With Concomitant Tuberculosis. Ann Thorac Surg. 2020;109(2):e119-e21.

100. Sekgololo JM, Frank CR, Moinuddeen V, Alireza DD, Calvin KM. Tuberculous aortitis as a rare cause of aortobronchial fistula with massive haemoptysis: A case report. International Journal of Surgery Case Reports. 2020;70:238-42.

101. Vijayvergiya R, Kasinadhuni G, Revaiah PC, Lal A, Sharma A, Kumar R. Thoracic endovascular aortic repair for aortobronchial fistula: a case series. Eur Heart J Case Rep. 2020;4(6):1-6.

102. Bounssir A, Jedar A, Azghari A, Bouhdadi H, Bakkali T, Lekehal B. TEVAR for thoracic mycotic aneurysm: Case report. Int J Surg Case Rep. 2021;81:105753.

103. Buerger M, Kapahnke S, Omran S, Schomaker M, Rief M, Greiner A, et al. Aortic aneurysm and aortic graft infection related to Mycobacterium bovis after intravesical Bacille Calmette-Guérin therapy-a case series. BMC Surg. 2021;21(1):138.

104. Fama’ F, Sindoni A, Donato R, Cascio A, Mondello P, Gaeta R. Tuberculous aortitis in an human immunodeficiency virus–positive Ivorian migrant: A case report. International Journal of STD and AIDS. 2021.

105. Koterazawa S, Watanabe J, Uemura Y, Uegaki M, Shirahase T, Taki Y. A case of infectious thoracic aortic aneurysm after intravesical Bacillus Calmette-Guérin instillation therapy for a superficial bladder cancer. Urol Case Rep. 2021;36:101574.

106. Luo P, Guo YY. Endovascular repair with stent-graft of symptomatic tuberculous aortic pseudoaneurysm. Annals of Vascular Surgery. 2021;23:23.

107. Velu D, Ibrahim F, Hassoun A. Tuberculous mycotic aneurysm of aortic arch: a case report emphasising on multidisciplinary management approach. BMJ Case Rep. 2021;14(2).
